# Supplementary material for: Quality of the diagnostic process in patients presenting with symptoms suggestive of bladder or kidney cancer: a systematic review
Source: BMJ Open. 2019 Oct 3;9(10):e029143. doi: 10.1136/bmjopen-2019-029143 (PMC6797416; doi:10.1136/bmjopen-2019-029143)
Supplement: Supplementary data [file bmjopen-2019-029143supp001.pdf]

Appendix 1 – Example Search Strategy (1st January 2000 to 28th January 2018)

1. exp Urinary Tract Infections/ or exp lower urinary tract symptoms/ or exp hematuria/ or exp polyuria/ or exp nocturia/ or h?ematuri*.ti,ab. Or dysuri*.ti,ab. Or (nocturi* or nycturi*).ti,ab. Or polyuri*.ti,ab. Or exp Urinary Incontinence/ or (urinary adj3 incontinen*).ti,ab. Or (urin* adj3 (frequen* or urgen*)).ti,ab. Or (urin* adj3 infect*).ti,ab. – 379274
2. exp Kidney Neoplasms/ or ((Kidney or renal) adj3 (cancer or neoplas* or tumo?r or carcinoma*)).ti,ab. – 213088
3. exp Urinary Bladder Neoplasms/ or (bladder adj3 (cancer or neoplas* or tumo?r or carcinoma*)).ti,ab. – 145154
4. #2 OR #3 – 346524
5. #1 AND #4 – 20778
6. limit #5 to (humans and yr="2000-Current") – 12950
7. limit #7 to "all adult (19 plus years)" [Limit not valid in Embase; records were retained] – 12268
8. limit #8 to ("all adult (19 plus years)" or "adolescent (13 to 18 years)") [Limit not valid in Embase; records were retained] – 12268
9. limit #6 to (adult <18 to 64 years> or aged <65+ years>) [Limit not valid in Ovid MEDLINE(R),Ovid MEDLINE(R) Daily Update,Ovid MEDLINE(R) In-Process,Ovid MEDLINE(R) Publisher; records were retained] – 8253

Cochrane Library Search Strategy (inception to 13th June 2019)

#1	MeSH descriptor: [Kidney Neoplasms] explode all trees	1046
#2	MeSH descriptor: [Urinary Bladder Neoplasms] explode all trees	1356
#3	MeSH descriptor: [Diagnosis] explode all trees	315302
#4	MeSH descriptor: [Urinary Tract Infections] explode all trees	2370
#5	MeSH descriptor: [Lower Urinary Tract Symptoms] explode all trees	2777
#6	MeSH descriptor: [Hematuria] 2 tree(s) exploded	177
#7	#1 OR #2	2371
#8	#4 OR #5 OR #6	5223
#9	#7 AND #8	37
#10	#9 AND #3	17
