# Supplementary material for: Quality of the diagnostic process in patients presenting with symptoms suggestive of bladder or kidney cancer: a systematic review
Source: BMJ Open. 2019 Oct 3;9(10):e029143. doi: 10.1136/bmjopen-2019-029143 (PMC6797416; doi:10.1136/bmjopen-2019-029143)
Supplement: Supplementary data [file bmjopen-2019-029143supp002.pdf]

Appendix 2: Reported percentages of diagnostic tests and urological referrals for patients with hematuria

Study	Type of hematuria[*]	No evaluation (%)	Frequency of different evaluations and referrals performed (%)							
			History/ Exam	Urine Culture	Urine Cytology	Imaging	Cystoscopy	Both Imaging and cystoscopy	Referral	Guideline concordant evaluation
**Performed within 30 days**										
**Matulewicz**	NVH					7.7	3.6	1.4		
**Performed within 60 days**										
**Richards 2018**	NVH	66								
**Richards 2018**	VH	67			-					
**Murphy**	VH[†]	47								
**Neider**	NVH			57-60	6-11	20-25			36	
**Performed within 90 days**										
**Richards 2018**	NVH	66								
**Richards 2018**	VH	47								
**Neider**	VH			40-56	5-13	38-41			69-77	
**Performed within 180 days**										
**Bassett**	NVH	65						14	23	
**Matulewicz**	NVH					14.3	8.9	5.1		
**Ark**	Either	54				44	20	18	35	
**Shinagare**	Either	36			43	76	35	64		36[‡]
**Friedlander**	Either					13.9	13.7	5.7		
**Performed within 365 days**										
**Matulewicz**	NVH					16.5	9.8	5.9		
**Performed within unspecified timescale (1-3 years)**										
**Elias**	NVH	42		15	10	23			21	
**Buteau**	Either	16		63	7.3					
**Bradley**	NVH		63	25						89[§]

**(secondary care)**										
**Buteau**	NVH			59	5	34	6	5		
**Buteau**	VH			84	20	42	26	25		
**Matulewicz**	NVH					6.2	3.0	2.5		

[*]NVH: non-visible hematuria; VH: visible hematuria
[+]Murphy included patients with 50 red blood cell per high power field (RBC/HPF), equivalent to very high risk and VH group
Definition of guideline concordant evaluation:
[‡] Shinagare: upper renal tract imaging, urine cytology and cystoscopy for high risk patients and 1 urinalysis with >3 RBC/HPF; or upper renal tract imaging followed by either urine cytology or cystoscopy for patients with no risk factors and 2 of 3 urinalysis with >3RBC/HPF.
[§] Bradley: cystoscopy and upper tract imaging using CT urography
