# Supplementary material for: Quality of the diagnostic process in patients presenting with symptoms suggestive of bladder or kidney cancer: a systematic review
Source: BMJ Open. 2019 Oct 3;9(10):e029143. doi: 10.1136/bmjopen-2019-029143 (PMC6797416; doi:10.1136/bmjopen-2019-029143)
Supplement: Author's manuscript [file bmjopen-2019-029143.draft_revisions.pdf]

BMJ Open

BMJ Open is committed to open peer review. As part of this commitment we make the peer review history of every article we publish publicly available.

When an article is published we post the peer reviewers' comments and the authors' responses online. We also post the versions of the paper that were used during peer review. These are the versions that the peer review comments apply to.

The versions of the paper that follow are the versions that were submitted during the peer review process. They are not the versions of record or the final published versions. They should not be cited or distributed as the published version of this manuscript.

BMJ Open is an open access journal and the full, final, typeset and author-corrected version of record of the manuscript is available on our site with no access controls, subscription charges or pay-per-view fees (http://bmjopen.bmj.com).

If you have any questions on BMJ Open's open peer review process please email
info.bmjopen@bmj.com

BMJ Open

Quality of the Diagnostic Process in Patients Presenting with Symptoms Suggestive of Bladder or Kidney Cancer: A Systematic Review

Journal:	*BMJ Open*
Manuscript ID	bmjopen-2019-029143
Article Type:	Research
Date Submitted by the Author:	14-Jan-2019

Complete List of Authors:	Zhou, Yin; University of Cambridge School of Clinical Medicine, Public Health and Primary Care<br>van Melle, Marije; University of Cambridge Department of Public Health and Primary Care<br>Singh, Hardeep; Michael E. DeBakey Veterans Affairs Medical Center, Houston Veterans Affairs Center for Innovations in Quality, Effectiveness and Safety; Baylor College of Medicine, Department of Medicine<br>Hamilton, Willie; University of Exeter Medical School, Primary Care Diagnostics<br>Lyratzopoulos, Georgios ; University College London, Department of Epidemiology & Public Health, Health Behaviour Research Centre<br>Walter, Fiona; University of Cambridge, Dept of Public Health and Primary Care

Keywords:	Urological tumours < UROLOGY, PRIMARY CARE, Health & safety < HEALTH SERVICES ADMINISTRATION & MANAGEMENT

SCHOLARONE™
Manuscripts

Quality of the Diagnostic Process in Patients Presenting with Symptoms Suggestive of Bladder or Kidney Cancer: A Systematic Review

Yin Zhou, MSc, MRCGP[1] (ykz21@medschl.cam.ac.uk)

Marije van Melle, PhD[1] (mav41@medschl.cam.ac.uk)

Hardeep Singh, MD, MPH[2] (hardeeps@bcm.edu)

William Hamilton, MD, FRCGP[3] (w.hamilton@exeter.ac.uk)

Georgios Lyratzopoulos, MD, FFPH, MPH[1,4] (y.lyratzopoulos@ucl.ac.uk)

Fiona M Walter, MD, FRCGP[1] (fmw22@medschl.cam.ac.uk)

[1] The Primary Care Unit, Department of Public Health and Primary Care, University of Cambridge, UK

[2] Center for Innovations in Quality, Effectiveness and Safety, Michael E. DeBakey VA Medical Center, and Department of Medicine, Baylor College of Medicine, Houston, Texas, USA

[3] University of Exeter Medical School, UK

[4] ECHO (Epidemiology of Cancer Healthcare & Outcomes) Research Group, Department of Behavioural Science & Health, University College London, UK

Corresponding author:

Yin Zhou ykz21@medschl.cam.ac.uk +44 (0)1223 748415

Word count: 3479
Abstract: 300
Tables: 4; **Figures:** 1; **Appendices:** 2
References: 45

For peer review only - http://bmjopen.bmj.com/site/about/guidelines.xhtml

ABSTRACT

Objectives

In urological cancers, sex disparity exists for survival, with women doing worse than men. Sub-optimal evaluation of presenting symptoms may contribute. We therefore examined factors affecting the quality of the diagnostic process for bladder or kidney cancer.

Methods

We searched Medline and Embase from 1 January 2000 to 29 January 2018 for studies that examined factors affecting the quality of the diagnostic process of patients presenting with symptoms of bladder or kidney cancer. Data extraction and quality assessment were independently performed by two authors. Due to the heterogeneity of study design and outcomes, the results could not be pooled. A narrative synthesis was performed.

We focused on one of the six domains of quality of health care described by the National Academy Sciences, Engineering and Medicine: timeliness. We also examined the quality of the diagnostic process more broadly, by assessing whether appropriate, or guideline-concordant, history, examination, tests and referrals were performed.

Results:

24 studies met review criteria, representing 106,731 people from 8 high-income countries. Studies were based in primary care, specialty care, or both (each n=8). Up to two-thirds of patients with haematuria received no further evaluation in the 6 months after their initial visit. Urinary tract infections, nephrolithiasis, and benign prostatic conditions before cancer diagnosis were associated with diagnostic delay. Women were more likely to experience

For peer review only - http://bmjopen.bmj.com/site/about/guidelines.xhtml

diagnostic delay than men. Patients who first saw a urologist were less likely to experience delayed evaluation and cancer diagnosis.

Conclusions:

Women, and patients with non-cancerous urological diagnoses just prior to their cancer diagnosis, were more likely to experience lower quality diagnostic processes. Using risk prediction tools and electronic algorithms to identify patients at risk of missed/delayed evaluation of symptoms, and improving guideline ambiguity on who should be actively followed-up, may improve outcomes and reduce sex disparity in survival for these cancers.

Strengths and Limitations of this study

- This is the first study to our knowledge that examined factors affecting the diagnostic quality of both kidney and bladder cancer.
- We examined all relevant symptoms in these patients, not limiting to haematuria only.
- We were unable to perform a meta-analysis due to the heterogeneity of the studies.

For peer review only

For peer review only - http://bmjopen.bmj.com/site/about/guidelines.xhtml

INTRODUCTION

Making a correct and timely diagnosis is paramount for patient safety and high quality healthcare. The United States (US) National Academy Sciences, Engineering and Medicine (NASEM- formerly the Institute of Medicine, IOM) report "Improving Diagnosis in Health Care" highlights the importance of research on reducing missed and delayed diagnosis and targeting contributory factors that lead to diagnostic errors [1]. Cancer is one of the commonest conditions to be affected by diagnostic errors [2] and outpatient malpractice claims [3]. This, in addition to the compelling rationale for early detection, makes cancer an excellent disease model for examining diagnostic safety. Bladder and kidney cancer, two relatively common cancers, pose particular diagnostic challenges. Uniquely among common cancers, women with bladder cancer have poorer survival than men with the same cancer [4]. Missed or delayed referral and diagnosis may contribute to the survival difference between men and women with these urological cancers [5].

Timeliness, one of the six domains of health care quality described by the NASEM, can be regarded as the most relevant for evaluating the diagnostic process in cancer [1]. Timely diagnosis of cancer is important to optimise clinical outcomes and patient experience [6,7]. In the United Kingdom (UK), efforts to promote early diagnosis and reducing delays during the diagnostic process have informed many initiatives aiming to improve outcomes for cancer patients [8].

We performed a systematic review to examine the factors affecting the quality of the diagnostic process, in particular timeliness, for patients presenting with urological

For peer review only - http://bmjopen.bmj.com/site/about/guidelines.xhtml

symptoms that may be suggestive of kidney or bladder cancer. Our secondary aim was to examine existing definitions for timeliness of evaluation, referral and diagnosis for these patients.

For peer review only

For peer review only - http://bmjopen.bmj.com/site/about/guidelines.xhtml

METHODS

Search Strategy and Study Inclusion

We searched Ovid Medline and Embase for relevant studies from January 1st 2000 to January 29th 2018, with no restrictions on publication type or languages (Appendix 1). We restricted our search to studies published from 2000 due to prior knowledge that there were few early relevant studies.

We focused on clinical features listed in the English 2015 National Institute for Health and Care Excellence (NICE) guidelines for suspected cancer [9] in order to examine the population that are most likely to have cancer. We based our outcome measures of diagnostic timeliness on internationally accepted definitions of the diagnostic intervals: e.g. primary care interval = time from the patient's first presentation to a primary care practitioner (PCP), to referral [10,11]. We also examined the quality of the diagnostic process more broadly, by assessing whether appropriate or guideline-concordant history, examination, diagnostic tests and referrals were performed during the evaluation of symptoms.

All titles and abstracts were screened by YZ, with 10% of a random selection independently assessed by a second reviewer (MM). Both authors then independently assessed the full-text articles after screening of titles and abstracts. Consensus was sought from GL and FW where disagreements arose.

Inclusion Criteria:

- Studies describing the factors that affect the timeliness or quality of the assessment of the following clinical features in the context or bladder or kidney cancer:

For peer review only - http://bmjopen.bmj.com/site/about/guidelines.xhtml

- Urinary tract infections (UTI)
- Haematuria
- Lower urinary tract symptoms (including dysuria, urinary frequency, urgency, incontinence and nocturia)

Exclusion Criteria:

- Studies only describing population or patients under the age of 18
- Conference abstracts, correspondence, editorials, short reports and the grey literature
- Case reports or case series of less than 10 patients.

Data extraction and quality assessment

YZ and MM independently performed data extraction, using a data collection template, on study characteristics, diagnostic intervals, frequency of evaluations, and the patient, clinician and system factors affecting the diagnostic intervals and frequency of evaluations. Quality appraisal was performed using a modified version of the Critical Appraisal Skills Programme (CASP) checklist for cohort studies (Table 1 Footnote) [12]. Any disagreements were resolved by discussion with all members of the research team.

Data synthesis and analysis

We were unable to pool the results due to the heterogeneity of the study design and outcomes. A narrative synthesis was therefore performed.

For peer review only - http://bmjopen.bmj.com/site/about/guidelines.xhtml

RESULTS

Study characteristics

24 papers were included after full-text reviews (Figure 1). All studies were from high-income countries. These include 16 from the US, two from Australia, two from the UK, and one each from Sweden, Canada, Austria and Italy, and Germany and Austria (in one study). Five examined cancer patients with no pre-defined clinical features (four bladder, one both bladder and kidney), four focused on patients with haematuria (one of which included only visible haematuria), six examined bladder cancer patients with haematuria, and one focused on upper urothelial tract cancer patients with haematuria. Eight studies were carried out each in the primary care setting, in hospital and both (Table 1).

For peer review only

For peer review only - http://bmjopen.bmj.com/site/about/guidelines.xhtml

Quality of Diagnostic Process

Diagnostic timeliness

Fourteen of the 24 included studies described diagnostic intervals for patients with either urological symptoms or who had been diagnosed with bladder or kidney cancer (Table 2).

Definitions of timely evaluation, referral and diagnosis, were described in 11 studies. For time to first evaluation including cystoscopy, upper urinary tract imaging or urine cytology, Garg et al. used a threshold of 30 days [13], while two studies examined proportions of patients undergoing these tests within 60 [14] and 90 days [15]. The remaining studies used 180 days as time cut-offs for which they considered evaluation should be carried out [16-19]. Thresholds for referral was set at 90 days by one paper [20], while delays in cancer diagnosis were defined as greater than 90 days [21] and in 3 month increments up to 1 year [22,23].

Other Quality Dimensions

We found no standard definition for high quality care during the diagnostic process in any of the included studies. Most studies reported the frequency of appropriate or guideline-concordant diagnostic tests and referrals performed during diagnostic evaluation (Appendix 2). Studies examining the frequency of non-evaluation of haematuria reported this to be 47 – 81% within 60 days of initial symptom presentation [14,15], reducing to 36 – 65 % in studies by 180 days [16-19].

For peer review only - http://bmjopen.bmj.com/site/about/guidelines.xhtml

Ten studies reported the percentages of investigations and referrals performed in patients with haematuria [14-19,24-27], six of which also specified time-frames during which these evaluations should be completed [14-19] (Table 3). Four studies reported that only 5 – 25% of these patients received both imaging and cystoscopy (commonly defined as 'complete evaluation' by the studies) by 6 months of their first presentation with haematuria [16-18,25] and one reported this percentage to be 64% in their sample of 100 patients [19].

Studies reported variations in the percentages of patients with haematuria who received urine culture (15 – 84%), urine cytology (5 – 43%), imaging tests (14 – 76%), and cystoscopy (6 – 26%) during the diagnostic process, indicating that there were variations in how clinicians evaluate patients with haematuria. A larger proportion of patients with visible haematuria (VH) (14-25%) [17,25] received both imaging and cystoscopy than patients with non-visible haematuria (NVH) (5%) [25]. Between 21-36% of patients with haematuria received a urological referral [16,17,26], although a survey study from PCPs reported that about a third and two-thirds of them would refer patients with NVH and VH respectively [27].

One secondary care study reported non guideline-compliant practice in over a third of post-menopausal women with asymptomatic haematuria, with no documentation of full genitourinary examination with vaginal tissue quality and presence of prolapse [24].

Presenting symptoms

Eight of the 18 studies which extracted symptom information used coded information from routine or claims data [16,17,21,22,24-26,28], four used self-reported symptoms from questionnaire

For peer review only - http://bmjopen.bmj.com/site/about/guidelines.xhtml

and audit data [29-32], five studies performed direct record review [14,15,19,25,33], and one used coded information and record review [34].

Eight studies examined the direct association between the type of presenting symptoms and quality of the diagnostic process, the majority focusing on haematuria [15,19,20,22,27,32,33] and one on UTI [35]. No other presenting symptoms were examined. Bladder or kidney cancer patients presenting with haematuria were significantly less likely to have 3 or more consultations before a GP referral compared to those who did not present with haematuria (OR 0.29 CI 0.19-0.46, p<0.001 for bladder cancer; OR 0.64 CI 0.30-1.37, p=0.25 for kidney cancer) [32]. There was also a dose-dependent relationship between the number of haematuria visits and the likelihood of a urological referral (HR 5.18 and 7.66 for 2 and 3 visits respectively vs 1 visit, p<0.0001) [20], and a positive association between number of haematuria visits and diagnostic delay for bladder cancer patients [22].

VH predicted a shorter time to evaluation [22], a lower likelihood of incomplete evaluation [15,19], shorter time from GP referral to urology consultation [33], and a shorter time to diagnosis [22] than NVH.

Other recent diagnoses preceding cancer diagnosis

Between 20-61% of women and 15-35% of symptomatic men were treated or diagnosed with a UTI before being diagnosed with bladder cancer [21,22,29,30]. Women are also four times as likely as men to receive three or more courses of treatments for UTIs before their cancer diagnosis (15.8% vs 3.8% for women vs men, p=0.04) [30].

For peer review only - http://bmjopen.bmj.com/site/about/guidelines.xhtml

In two case series it was also reported that a significant proportion of bladder cancer patients (up to 40%) received symptomatic treatments for either lower urinary tract symptoms or abdominal pain before referral to a urologist [29] or were not further evaluated, with women more likely to be affected than men (41.7% vs 16.2% once or twice, 5.6% vs 2.9% three or more times, women vs men; p=0.04) [30].

Two large US studies reported that benign diagnosis (up to 12 months prior to cancer diagnosis) such as UTIs, nephrolithiasis and prostate-related diagnosis were associated with delays in cancer diagnosis [21,22]. UTIs were associated with a two-fold increase in the odds of diagnostic delay by at least 3 months in both sexes for both upper tract urothelial cancer and bladder cancer [21,22], compared to those with no UTI diagnosis pre-diagnosis (OR 1.97, CI 1.74-2.22) [22]. This was regardless of whether patients first presented to a urologist or other specialty doctors [21]. Nephrolithiasis (RR 1.29, CI 1.07-1.54; p=0.007 vs RR 1.09, 0.81-1.47 for men vs women) and benign prostatic conditions (such as prostatitis, benign prostatic hyperplasia and benign prostatic nodule) were more likely to predict diagnostic delay in men than women with upper tract urothelial cancer [21]. In this nationwide insurance claims study, no validation of coded information was performed using medical record review.

Patient factors

Sex

The effect of sex on diagnostic activity and timeliness were reported by 12 studies [13,16,18-20,22,25,26,28-30,32]. Most evidence indicated that women were less likely than men to undergo any investigation [17], have complete evaluation [17], be referred to a specialist [20,28,33], and to

For peer review only - http://bmjopen.bmj.com/site/about/guidelines.xhtml

have cystoscopy or imaging [18,33]. Female sex was also a consistent predictor for delayed evaluation of haematuria [13,16,22] and UTIs [25,30], and longer diagnostic intervals for cancer [13,28,32]. This sex disparity with respect to evaluation and referral was insignificant for patients with NVH [19,25,26].

Other patient factors

In general, evidence for other patient factors affecting the quality of the diagnostic process was less consistent. Two large US studies consisting of over 65,000 patients in total found that older bladder cancer patients with haematuria had longer delays to evaluation than younger patients [13,23]. Increasing comorbidity predicted slower time to urologist, longer delay to evaluation [13], and diagnosis [22] in two large US samples. While five of the six studies reported no association between ethnicity and quality of the diagnostic process, one study with about 1400 participants reported that African-American bladder cancer patients were less likely than their Caucasian counterparts to: be referred to a urologist (adjusted OR 0.72; 95% CI 0.56-0.93; have a cystoscopy (adjusted OR 0.67; 95% CI 0.50-0.89), or have imaging (adjusted OR 0.75; 95% CI 0.59-0.95) [16].

The evidence between socioeconomic status, comorbidity, smoking, and anticoagulant use was either non-significant or weak (Table 3).

Clinician factors

Physician type

Six studies from the US examined the type of clinicians as a predictor for diagnostic delay (Table 4). Patients who first saw a urologist for their symptoms were less likely to have a

For peer review only - http://bmjopen.bmj.com/site/about/guidelines.xhtml

delay in evaluation [13] or cancer diagnosis [21] and more likely to have guideline-adherent evaluation [19] than those who first saw another specialty doctor.

When comparing specialties excluding urology, a mixed pattern was seen with respect to referral and use of investigations. In general, there was little evidence to suggest that family physicians in the US differ from other specialists with respect to evaluating haematuria. Family physicians may be less likely to refer for VH [20,25] although evidence for delayed referral in patients with UTI and NVH was less clear [25,27].

System factors

Diagnostic pathways

Two studies examined the impact of interventions in the diagnostic pathways on diagnostic intervals in the UK and Sweden respectively [31,36]. A single institution UK study found that the time from GP referral to first hospital visit shortened from 42.9 days to 21.3 days ($p<0.001$) after the introduction of the fast-track pathway, in which patients with alarm symptoms are typically seen or investigated by a specialist within two weeks of a GP referral [36]. In Sweden, the introduction of a telephone hotline for patients with VH reduced the time from haematuria to urology referral (33 to 14 days, $p=0.32$), referral to diagnosis (19 to 8 days, $p=0.003$), and total healthcare interval (50 to 29 days, $p=0.03$) [31]. Patients with eligible symptoms were able to access a nurse consultant directly by telephone, who then scheduled the patient for serum creatinine, urine cytology and appointment with a urologist for flexible cystoscopy and CT urography within 2 weeks, all with the same priority as other patients referred by their GP but bypassing the routine referral system [31].

For peer review only - http://bmjopen.bmj.com/site/about/guidelines.xhtml

Other factors

Other factors that were found to impact on the quality of the diagnostic process were described by studies using direct record review. These include patient factors such as not attending, cancelling or declining to attend follow up appointments [14], delays in PCPs reviewing results, lack of receipt of referral [34], and scheduling and coordination delay of follow-up test or appointment [14,15], although the detailed effects of these factors on the quality of the diagnostic process were not reported.

DISCUSSION

Our review identified several potential areas of missed opportunities in urological cancer diagnosis; it also provides evidence for informing the development of future interventions and research.

Non-evaluation of haematuria

Studies reported high frequencies of non-evaluation of haematuria, with about two-thirds of patients having no evaluation up to 180 days after initial presentation. Although we found no consistent definition of diagnostic timeliness for evaluation, referral and diagnosis of urological cancer, high percentages of non-evaluated cases likely harbor missed opportunities for a timely diagnosis. For instance, patients with VH should receive renal function testing, imaging and urology referral for cystoscopy once a transient cause such as UTI has been excluded [37]. Patients with persistent NVH should additionally receive a blood pressure check and urinary albumin-creatinine ratio as part of the evaluation [37]. Given that the PPV of haematuria for urological cancer can be as high as 11% [38], lack of evaluation

For peer review only

could lead to missed diagnoses. Improving clinicians' awareness and adherence to existing guidelines as well as using electronic algorithms to flag up abnormal findings [14] may reduce such missed opportunities [39].

Women experience poorer quality of diagnostic process than men

Our review found that women with haematuria were more likely to be treated for UTIs or for pain, and less likely to be evaluated further or referred than men. Benign conditions such as UTIs and atrophic vaginitis are commoner in women. At the same time women are less likely to have bladder and kidney cancer than men (M: F ratio about 3:1 and 1.7:1 for bladder and kidney respectively) [40]. This may be due to differing exposure to lifestyle and environmental factors, and biological propensity to these cancers by sex [4]. The combined effect of greater frequency of non-neoplastic disease in women, and the fact that urological cancer is less common in women, means that the PPV of relevant symptoms for bladder and kidney cancer is lower in women than men.

Although a relevant urological symptom is more likely to be due to a benign cause in women than in men, avoidable diagnostic delay for urological cancer may still occur if there is a failure to ensure the resolution of symptoms. Current American Urological Association (AUA) guidelines on the evaluation of asymptomatic microscopic haematuria recommends repeat urinalysis after the treatment of other causes, and subsequent renal function testing, cystoscopy and imaging if symptoms do not resolve after treatment [41]. However, the low diagnostic yield of NVH evaluation and the frequency of other benign causes (such as infection) in everyday clinical practice may affect guideline adherence, and contribute to diagnostic delay. Future research should examine risk stratification based not only on

For peer review only - http://bmjopen.bmj.com/site/about/guidelines.xhtml

presence of symptoms, but also on their severity, chronicity, recurrence or persistent nature. For example, guidelines should address cut-offs for degree of NVH that should warrant active reviews after treatments for UTIs; or cut-offs for number of UTIs treated before referral), while also taking into account cost-effectiveness of any follow-up actions. Emerging urine biomarkers and risk prediction tools may also be useful additions to improve diagnostic yield [42,43].

Concomitant benign conditions

The challenge in clinical practice arises when a presenting symptom may be the result of concomitant benign disease or cancer. In these cases it is likely that a significant proportion of patients will be appropriately treated and reviewed, averting unnecessary investigations.

The observation that a UTI diagnosis delays cancer diagnosis in patients first seen by both urologists and non-urologists [21] suggests that diagnostic reasoning is challenging for clinicians in such situations. The PPV of a symptom for cancer probably falls if the patient has concomitant diseases which cause the symptom. Some evidence suggests that conditions such as benign prostate disease and kidney stones also delay the diagnosis of kidney cancer [21]. Whether and how much of this diagnostic delay is avoidable is yet to be determined, and should be a priority for future research. While current UK guidelines recommend that patients with persistent or recurrent UTIs should be referred for further evaluation, there is no US equivalent, nor guidelines on the management of other concomitant benign conditions and possible cancer. When no guideline exists, or adherence is not possible, close follow-up to ensure improvement or resolution of symptoms should take place, and patients should be instructed to return if symptoms do not improve.

For peer review only - http://bmjopen.bmj.com/site/about/guidelines.xhtml

Clinician and system factors

Patients experienced shorter diagnostic intervals if they first presented to a urologist instead of another specialty doctor. This is not surprising given that urologists are likely to have better access to investigations, typically consisting of cystoscopy and upper renal tract imaging. The variations seen in evaluation and referral between other clinical specialties may indicate different levels of guideline awareness and adherence, although this evidence is scarce and inconsistent.

Process or system delays, such as patient non-attendance at appointments, delays in scheduling of appointments or non-receipt of referrals, all contribute to diagnostic delay [14,15,34,44], although the magnitude of their effects is unclear. In addition to improving process and workflow issues within primary and secondary care services, wider system changes such as providing direct access to imaging and streamlining referral processes may also play a role in expediting cancer diagnosis. Diagnostic pathways such as the fast-track referral system for patients with alarm symptoms in the UK, or a telephone hotline service, may shorten primary care and total healthcare interval, although the cost-effectiveness of such pathways need to be evaluated in specific health system contexts.

Strengths and limitations

Our review is the first to examine the evidence relating to factors affecting the quality of the diagnostic process in patients with bladder and kidney cancer. It builds on a previous review examining haematuria assessment in bladder cancer patients [45], and looks at a range of urological symptoms, and also patients with kidney cancer.

For peer review only - http://bmjopen.bmj.com/site/about/guidelines.xhtml

Unfortunately, we were unable to perform a meta-analysis due to the heterogeneity in study designs and outcomes. We were also unable to check the veracity of the co-morbid disease labels in the papers that used coded information. All the studies are from high-income countries, and therefore may be less generalizable to other countries with differing health care systems.

Conclusion

We found lack of consistency in defining diagnostic quality, including timeliness, of bladder and kidney cancer, and insufficient exploration of population-based evidence related to clinician and system factors affecting the quality of the diagnostic process. Our review highlights the need to improve evaluation of haematuria, and to develop high-quality evidence to inform guidelines on specific thresholds for active follow-up of high-risk symptomatic patients, which could be incorporated into risk prediction tools and clinical decision support. Future research should also identify and target preventable delays, especially in the context of concomitant benign conditions. Identifying patients with evaluation delays through electronic algorithms may also improve outcomes and reduce the sex inequality in survival for these cancers. In sum, our review identifies several potential areas of missed opportunities in bladder and kidney cancer diagnosis that may be avoidable and amenable to targeted interventions.

For peer review only - http://bmjopen.bmj.com/site/about/guidelines.xhtml

For peer review only

Acknowledgements

The authors would like to thank Isla Kuhn, medical librarian at the University of Cambridge School of Clinical Medicine Medical Library, for her advice and assistance with the development of the strategy strategy.

Funding

YZ is supported by a Wellcome Trust Primary Care Clinician PhD Fellowship (203921/Z/16/Z). The authors WH and FW are co-Principal Investigators and the authors YL and HS are co-Investigators of the multi-institutional CanTest Research Collaborative funded by a Cancer Research UK Population Research Catalyst award (C8640/A23385). HS is additionally supported by the Houston VA HSR&D Center for Innovations in Quality, Effectiveness and Safety (CIN 13-413).

All authors declare no conflicts of interest.

Author contribution

YZ, GL and FW designed the study. YZ developed and performed the search with the help of Isla Kuhn, medical librarian at the University of Cambridge. YZ and MM performed the data extraction with MM. YZ drafted the manuscript, MM, HS, WH, GL and FW critically revised the article.

For peer review only - http://bmjopen.bmj.com/site/about/guidelines.xhtml

References

Uncategorized References

1. Institute of Medicine. Improving diagnosis in health care. 2015; https://www.nap.edu/catalog/21794/improving-diagnosis-in-health-care. Accessed August 26th 2018.
2. Kostopoulou O, Delaney BC, Munro CW. Diagnostic difficulty and error in primary care—a systematic review. *Family practice.* 2008;25(6):400-413.
3. Gandhi TK, Kachalia A, Thomas EJ, et al. Missed and delayed diagnoses in the ambulatory setting: a study of closed malpractice claims. *Annals of internal medicine.* 2006;145(7):488-496.
4. Dobruch J, Daneshmand S, Fisch M, et al. Gender and bladder cancer: a collaborative review of etiology, biology, and outcomes. *European urology.* 2016;69(2):300-310.
5. Neal R, Tharmanathan P, France B, et al. Is increased time to diagnosis and treatment in symptomatic cancer associated with poorer outomes? Systematic review. *British journal of cancer.* 2015;112:S92-S107.
6. Mendonca SC, Abel GA, Saunders CL, Wardle J, Lyratzopoulos G. Pre‐referral general practitioner consultations and subsequent experience of cancer care: evidence from the English Cancer Patient Experience Survey. *European Journal of Cancer Care.* 2015;25(3):478-490.
7. Hamilton W, Walter FM, Rubin G, Neal RD. Improving early diagnosis of symptomatic cancer. *Nature Reviews Clinical Oncology.* 2016;13(12):740-749.
8. Hiom S. Diagnosing cancer earlier: reviewing the evidence for improving cancer survival. *British journal of cancer.* 2015;112:S1-S5.
9. NICE. NICE guidelines [NG12]: Suspected cancer: recognition and referral. 2015; http://www.nice.org.uk/guidance/NG12/. Accessed 1st September 2018.
10. Walter F, Webster A, Scott S, Emery J. The Andersen Model of Total Patient Delay: a systematic review of its application in cancer diagnosis. *Journal of health services research & policy.* 2012;17(2):110-118.
11. Weller D, Vedsted P, Rubin G, et al. The Aarhus statement: improving design and reporting of studies on early cancer diagnosis. *British Journal of Cancer.* 2012;106(7):1262-1267.
12. Critical Appraisal Skills Programme. CASP Cohort Study Checklist. 2017; http://www.casp-uk.net/checklists. Accessed 1st September 2018.
13. Garg T, Pinheiro LC, Atoria CL, et al. Gender disparities in hematuria evaluation and bladder cancer diagnosis: a population based analysis. *Journal of Urology.* 2014;192(4):1072-1077.
14. Murphy D, Meyer A, Vaghani V, et al. Application of Electronic Algorithms to Improve Diagnostic Evaluation for Bladder Cancer. *Applied clinical informatics.* 2017;8(1):279.
15. Richards KA, Ruiz VL, Murphy DR, et al. Diagnostic evaluation of patients presenting with hematuria: An electronic health record-based study. *Urologic Oncology: Seminars and Original Investigations.* 2018;36(3):88.e19-88.e25.
16. Ark JT, Alvarez JR, Koyama T, et al. Variation in the Diagnostic Evaluation among Persons with Hematuria: Influence of Gender, Race and Risk Factors for Bladder Cancer. *Journal of Urology.* 2017;198(5):1033-1038.

For peer review only - http://bmjopen.bmj.com/site/about/guidelines.xhtml

17. Bassett JC, Alvarez J, Koyama T, et al. Gender, race, and variation in the evaluation of microscopic hematuria among Medicare beneficiaries. *Journal of General Internal Medicine.* 2015;30(4):440-447.
18. Friedlander DF, Resnick MJ, You C, et al. Variation in the intensity of hematuria evaluation: a target for primary care quality improvement. *American Journal of Medicine.* 2014;127(7):633-640.e611.
19. Shinagare AB, Silverman SG, Gershanik EF, Chang SL, Khorasani R. Evaluating hematuria: Impact of guideline adherence on urologic cancer diagnosis. *American Journal of Medicine.* 2014;127(7):625-632.
20. Johnson EK, Daignault S, Zhang Y, Lee CT. Patterns of hematuria referral to urologists: does a gender disparity exist? *Urology.* 2008;72(3):498-502; discussion 502-493.
21. Chappidi MR, Kates M, Tosoian JJ, et al. Evaluation of gender-based disparities in time from initial haematuria presentation to upper tract urothelial carcinoma diagnosis: analysis of a nationwide insurance claims database. *BJU International.* 2017;120(3):377-386.
22. Cohn JA, Vekhter B, Lyttle C, Steinberg GD, Large MC. Sex disparities in diagnosis of bladder cancer after initial presentation with hematuria: a nationwide claims-based investigation. *Cancer.* 2014;120(4):555-561.
23. Hollenbeck BK, Dunn RL, Ye Z, et al. Delays in diagnosis and bladder cancer mortality. *Cancer.* 2010;116(22):5235-5242.
24. Bradley MS, Willis-Gray MG, Amundsen CL, Siddiqui NY. Microhematuria in Postmenopausal Women: Adherence to Guidelines in a Tertiary Care Setting. *Journal of Urology.* 2016;195(4 Pt 1):937-941.
25. Buteau A, Seideman CA, Svatek RS, et al. What is evaluation of hematuria by primary care physicians? Use of electronic medical records to assess practice patterns with intermediate follow-up. *Urologic Oncology: Seminars and Original Investigations.* 2014;32(2):128-134.
26. Elias K, Svatek RS, Gupta S, Ho R, Lotan Y. High-risk patients with hematuria are not evaluated according to guideline recommendations. *Cancer.* 2010;116(12):2954-2959.
27. Nieder AM, Lotan Y, Nuss GR, et al. Are patients with hematuria appropriately referred to Urology? A multi-institutional questionnaire based survey. *Urologic Oncology.* 2010;28(5):500-503.
28. Santos F, Dragomir A, Kassouf W, Franco E, Aprikian A. Urologist referral delay and its impact on survival after radical cystectomy for bladder cancer. *Current Oncology.* 2015;22(1):e20-e26.
29. Aziz A, Madersbacher S, Otto W, et al. Comparative analysis of gender-related differences in symptoms and referral patterns prior to initial diagnosis of urothelial carcinoma of the bladder: a prospective cohort study. *Urologia Internationalis.* 2015;94(1):37-44.
30. Henning A, Wehrberger M, Madersbacher S, et al. Do differences in clinical symptoms and referral patterns contribute to the gender gap in bladder cancer? *BJU International.* 2013;112(1):68-73.
31. Liedberg F, Gerdtham U, Gralen K, et al. Fast-track access to urologic care for patients with macroscopic haematuria is efficient and cost-effective: results from a prospective intervention study. *British Journal of Cancer.* 2016;115(7):770-775.

For peer review only - http://bmjopen.bmj.com/site/about/guidelines.xhtml

32. Lyratzopoulos G, Abel GA, McPhail S, Neal RD, Rubin GP. Gender inequalities in the promptness of diagnosis of bladder and renal cancer after symptomatic presentation: Evidence from secondary analysis of an English primary care audit survey. *BMJ Open.* 2013;3(e002861).
33. Ngo B, Papa N, Perera M, Bolton D, Sengupta S. Predictors of delay to cystoscopy and adequacy of investigations in patients with haematuria. *BJU International.* 2017;119 Suppl 5:19-25.
34. McCombie SP, Bangash HK, Kuan M, Thyer I, Lee F, Hayne D. Delays in the diagnosis and initial treatment of bladder cancer in Western Australia. *BJU International.* 2017;120 Suppl 3:28-34.
35. Richards KA, Ham S, Cohn JA, Steinberg GD. Urinary tract infection-like symptom is associated with worse bladder cancer outcomes in the Medicare population: Implications for sex disparities. *International Journal of Urology.* 2016;23(1):42-47.
36. Blick C, Bailey D, Haldar N, Bdesha A, Kelleher J, Muneer A. The impact of the two-week wait rule on the diagnosis and management of bladder cancer in a single UK institution. *Annals of the Royal College of Surgeons of England.* 2010;92(1):46-50.
37. Kelly JD, Fawcett DP, Goldberg LC. Assessment and management of non-visible haematuria in primary care. *BMJ: British Medical Journal (Online).* 2009;338(a3021).
38. Shapley M, Mansell G, Jordan JL, Jordan KP. Positive predictive values of ⩾ 5% in primary care for cancer: systematic review. *Br J Gen Pract.* 2010;60(578):e366-e377.
39. Murphy DR, Wu L, Thomas EJ, Forjuoh SN, Meyer AN, Singh H. Electronic trigger-based intervention to reduce delays in diagnostic evaluation for cancer: a cluster randomized controlled trial. *Journal of Clinical Oncology.* 2015;33(31):3560.
40. Smittenaar C, Petersen K, Stewart K, Moitt N. Cancer incidence and mortality projections in the UK until 2035. *British Journal of Cancer.* 2016;115(9):1147-1155.
41. American Urological Association. Diagnosis, Evaluation and Follow-up of Asymptomatic Microhematuria (AMH) in Adults. 2012; http://www.auanet.org/guidelines/asymptomatic-microhematuria-(2012-reviewed-for-currency-2016). Accessed 1st September 2018.
42. Loo RK, Lieberman SF, Slezak JM, et al. Stratifying risk of urinary tract malignant tumors in patients with asymptomatic microscopic hematuria. *Mayo Clinic Proceedings.* 2013;88(2):129-138.
43. Tan WS, Ahmad A, Feber A, et al. Development and validation of a haematuria cancer risk score to identify patients at risk of harbouring cancer. *Journal of Internal Medicine.* 2018;doi: 10.1111/joim.12868.
44. Weingart SN, Stoffel EM, Chung DC, et al. Delayed workup of rectal bleeding in adult primary care: examining process-of-care failures. *The Joint Commission Journal on Quality and Patient Safety.* 2017;43(1):32-40.
45. Ngo B, Perera M, Papa N, Bolton D, Sengupta S. Factors affecting the timeliness and adequacy of haematuria assessment in bladder cancer: a systematic review. *BJU International.* 2017;119 Suppl 5:10-18.

For peer review only - http://bmjopen.bmj.com/site/about/guidelines.xhtml

For peer review only

For peer review only - http://bmjopen.bmj.com/site/about/guidelines.xhtml

Table 1: Study characteristics of included publications and quality assessment

Papers	Year/s of data collection	Design	Setting (primary care, specialist or both)	Sample size	Population	CASP Quality Assessment Items A	B	C	D	E	F	G	H	I
Ark 2017, USA	2001-2009	Retrospective cohort study	Primary care	1412	Patients aged 40+ with bladder cancer and haematuria	😊	😊	😊	😊	😊	😊	😊	😊	😊
Aziz 2015, Germany & Austria	2010-2013	Retrospective questionnaire	Specialist	68	Patients undergoing TUR-BT* for newly diagnosed urothelial carcinoma of bladder	😐	☹️	😐	☹️	☹️	☹️	☹️	☹️	😐
Bassett 2015, USA	2009-2010	Retrospective cohort study	Primary care	9211	Patients with non-visible haematuria seen by a PCP† in an outpatient setting, age 65+	😊	😊	😊	😊	😊	😊	😊	😊	😊
Blick 2010, UK	1997-2006	Retrospective record review	Specialist	200	Consecutive patients with newly diagnosed bladder cancer identified from the records of MDT‡ meetings and theatre records	😊	☹️	☹️	😊	☹️	☹️	☹️	☹️	😊
Bradley 2016, USA	2012-2014	Retrospective record review/ cross-sectional cohort	Specialist	237	Women >55 with asymptomatic micro-haematuria	😊	😊	😊	😐	😐	☹️	😐	☹️	😊
Buteau 2014, USA	2009-2010	Retrospective record review	Primary care	449	Patients with over 5RBC/HPF§, with both gross and microscopic haematuria	😊	😊	😊	😊	😊	☹️	😐	☹️	😊
Chappidi 2017, USA	2010-2014	Retrospective claims review	Both	1326	Patients with UTUC** with a haematuria claim 1 year before diagnosis, under 65, 4.4% (n=58) with concomitant bladder cancer diagnosis at time of UTUC diagnosis	😊	😊	😊	😊	😊	😊	😊	😊	😊
Cohn 2014, USA	2004-2010	Retrospective claims review	Both	7649	Patients who had an initial haematuria claim within 12 months of an initial bladder cancer claim, age < 66 (upper limit of MarketScan Database population)	😊	😊	😊	😊	😊	😊	😊	😊	😊
Elias 2010, USA	2006-2007	Retrospective record review	Primary care	164	Participants with microscopic haematuria on urine dipstick testing or 3+RBC/HPF on urinalysis. Recruited from well patient clinics aged 50+ with a 10-year or greater smoking history (any number of cigarettes) and/or a significant (15 or more years) high-risk occupation (such as working in the dye, petroleum, or chemical industry.	😊	😊	😊	😊	😊	😊	😊	😊	😊
Friedlander 2014, USA	2004-2012	Retrospective cohort review	Primary care	2455	Patients aged 40+ with first episode of haematuria between 2004-2012 either by urinalysis (>3RBC/HPF) or ICD diagnosis codes for haematuria	😊	😊	😐	😊	😊	😊	😐	😐	😊
Garg 2014, USA	2000-2007	Retrospective cohort study	Both	35646	Patients aged 66+ with primary bladder cancer with a haematuria claim in 12 months before diagnosis	😊	😊	😊	😊	😊	😊	😊	😊	😊

Henning 2013, Austria & Italy	Not mentioned	Prospective questionnaire study	Specialist	200	Consecutive series of 200 patients admitted for elective TUR-BT	☺	☺	☹	😐	☹	☹	😐	😐	☺
Hollenbeck 2010, USA	1992-2002	Retrospective cohort study	Both	29740	Patients with haematuria 1 year prior to bladder cancer diagnosis; 66+	☺	☺	☺	☺	☺	☹	😐	😐	☺
Johnson 2008, USA	1998-2002	Retrospective cohort study	Both	926	Patients with newly diagnosed haematuria, 18+	☺	😐	☺	😐	😐	😐	☺	☺	☺
Liedberg 2016, Sweden	2014-2015	Interventional study	Both	275	Patients in intervention group, 101 in control group; patients=50+ with macroscopic haematuria	☺	😐	😐	☺	☹	☹	😐	😐	☺
Lyratzopoulos 2013, UK	2009-2010	Secondary analysis of audit data	Primary care	1318	Bladder and kidney cancer patients	☺	☺	☺	☺	☺	☺	☺	☺	☺
McCombie 2017, Australia	2008-2014	Retrospective cohort study, telephone survey	Specialist	100	Bladder cancer patients	☺	☺	😐	☺	☹	☹	😐	☹	☺
Murphy 2017, USA	2012-2014	Retrospective cohort study	Primary care	495	Patients with haematuria (urine red blood cells of >50 cells per high power field)	☺	😐	😐	☺	☹	☹	😐	😐	☺
Ngo 2017, Australia	2015 (12 month period)	Retrospective record review	Specialist	305	Cystoscopy cases primarily for haematuria investigation, 18+	☺	☺	☺	☺	☺	☺	☺	😐	☺
Nieder 2010, USA	Not mentioned	Questionnaire study	Primary care	788	788 PCPs (internal medicine, family practice, primary care, or obstetrics and gynaecology) randomly selected from the Little Blue Book of Miami-Dade County and Dallas, published by National Physicians Data Source	☺	☺	😐	☹	☹	☹	😐	😐	☺
Richards 2016, USA	2007-2009	Retrospective cohort study	Both	12195	Patients from SEER-Medicare with a haematuria or UTI[††] claim; 66+	☺	☺	☺	☺	☺	☺	☺	☺	☺
Richards 2018, USA	2011-2013	Retrospective record review	Both	201	Consecutive patients with new-onset haematuria, 195 male	☺	😐	☺	☺	😐	☺	☺	😐	☺
Santos 2015, Canada	2000-2009	Retrospective cohort review	Specialist	1271	Patients who underwent radical cystectomy for bladder cancer, had a first urologist visit after having visited a GP[‡‡] or ED[§§] physician, >40+ age	☺	😐	😐	😐	😐	☹	😐	☹	☺
Shinagare 2014, USA	2004-2012	Retrospective record review	Specialist	100	Consecutive patients with asymptomatic haematuria	☺	😐	☺	☺	☹	😐	😐	☹	😐

Footnotes
[*] TUR-BT: transurethral resection of bladder tumour
[†] PCP: primary care practitioner
[‡] MDT: multidisciplinary team
[§] RBC/HPF: red blood cell per high power field
[**] UTUC: upper tract urothelial carcinoma
[††] UTI: urinary tract infection
[‡‡] GP: general practitioner
[§§] ED: emergency department

CASP Quality Assessment Items (Key: green=yes, yellow=can't tell, red=no)
A: Does the study address a clearly focused issue?
B: Was the cohort recruited in an acceptable way?
C: Was the exposure accurately measured to minimise bias?
D: Was the outcome accurately measured to minimise bias?
E: Have the authors identified all important confounding factors?
F: Have they taken account of the confounding factors in the design and/or analysis?
G: Do you believe the results?
H: Can the results be applied to the local population?
I: Do the results of this study fit with the other available evidence?

For peer review only

Table 2: Mean and median diagnostic intervals from first presentation to diagnosis as described in included studies

Study	Population of interest*	Time interval ‡	Event points (shading denotes interval): First presentation with symptom	Referral to specialist	First specialist appointment	First investigation	Diagnosis	Mean interval duration (days): All	Men	Women	Median interval duration (days): All	Men	Women
**Aziz 2015, Austria/ Germany**	VH⁺	T1						335	343.70	313.29			
**Chappidi 2017, USA**	UTUC⁺ with haematuria	T1							93.5	84.4		60	49
**Cohn 2014, USA**	Bladder cancer with haematuria	T1							85.4	73.6		41	35
**Liedberg 2016, Sweden**	VH⁺	T1									29 vs 50	(control vs intervention)	
**Richards 2016, USA**	Bladder	T1							58.9	72.2			
**Garg 2014, USA**	Bladder cancer with haematuria	T1									8	8	9
**Garg 2014, USA**	Bladder cancer with haematuria	T2						27	24	35	3	2	6
**Richards 2018, USA**	VH⁺	T3									23		
**Richards 2018, USA**	NVH⁺	T3									28		
**Santos 2015, USA**	Bladder	T3							30	23	56		
**Chappidi 2017, USA**	UTUC⁺ with haematuria	T3							17.9	20.4		4	5
**Johnson 2008, USA**	Haematuria	T4									33.5	27.4	36.5
**Liedberg 2016, Sweden**	VH⁺	T4									14 vs 33	(control vs intervention)	
**Lyratzopoulos 2013, UK**	Bladder	T4										4	6
**Lyratzopoulos 2013, UK**	Kidney	T4										10	16
**McCombie 2017, Australia**	Bladder	T4									3		
**Liedberg 2016, Sweden**	VH^	T5									8 vs 19	(control vs intervention)	
**Blick 2010, UK**	Bladder	T6						21.3					
**McCombie 2017, Australia**		T6									23.5		
**McCombie 2017, Australia**		T6a									3		
**McCombie 2017, Australia**		T6b									19.5		
**Ngo 2017 Australia**	Haematuria	T6									38		
**Ngo 2017 Australia**	Haematuria	T7									28		

*** Population of interest indicates the characteristics of the cohort examined in each study (either symptom or type of cancer patients)**
⁺ VH = visible haematuria; NVH = non-visible haematuria; UTUC: upper tract urothelial carcinoma
‡ Key for time intervals:

T1: Time from first presentation with symptom to diagnosis
T2: Time from first presentation with symptom to first investigation
T3: Time from first presentation to first specialist appointment
T4: Time from first presentation to referral to specialist
T5: Time from referral to specialist to diagnosis
T6: Time from referral to specialist to first specialist appointment
T6a: Time from referral being sent to referral being received
T6b: Time from receipt of referral to first specialist appointment
T7: Time from first specialist appointment to first investigation

For peer review only

Table 3: Summary of association between patient factors and diagnostic safety and timeliness

Patient factor	Number of studies exploring risk factor	Association between patient factor and diagnostic safety and timeliness		
		Delayed / Incomplete Evaluation	Delayed Referral	Longer Diagnostic interval
Sex	12	Women>Men	Women>Men	Women >Men
Increasing age	10	NS [15,25,26]<br>Positive association [13,23]	NS [13,25,33]	NS [22]
Ethnicity	6	NS [13,17,18,23,25]<br>African-American worse [16]	NS [13,17,18,23,25]<br>African-American worse [16]	NS [13,17,18,23,25]
SES	5	NS [18,22,23,31,33]		
Comorbidity	4	Positive association [13]<br>NS [15,21]		Positive association [22]<br>NS [15,21]
Smoking	5	NS [17,18,25,33]<br>Positive association [26]	NS [17,18,25,33]	
Anticoagulant use	4	NS [15,17,33]<br>More likely to receive imaging [18]	NS [15,17,33]	
NS: statistically non-significant				

For peer review only

Table 4: Associations between physician specialty and quality of diagnostic process for patients presenting with different clinical features pre-cancer diagnosis

Clinical feature	Delay in evaluation	Delay in referral	Delay in diagnosis
**Urinary tract infection (UTI)**	No difference between PCP* and other specialists or ED+ physicians (Buteau)	No difference between PCP* and other specialists or ED+ physicians (Buteau)	Both urologist and non-urologist (Urologist: RR1.74, CI 1.31-2.31 p<0.001; non-urologist RR 1.44, CI 1.22-1.71 p<0.001 (Chappidi)
**Microscopic haematuria**	No difference between PCP* and other specialists or ED+ physicians (Buteau); OBGYN‡ less likely to perform imaging than medical counterparts (p<0.004) (Neider); guideline concordant with urologist vs non-urologist (OR 54.7, CI 10-102, p<0.0001) (Shinagare)	No difference between PCP* and other specialists or ED+ physicians (Buteau, Neider)	
**Macroscopic haematuria**	OBGYN‡ less likely to perform imaging than medical counterparts (p<0.01) (Neider)	PCP* less than other specialists or ED+ physicians (Buteau); no difference between specialties (Neider)	
**Haematuria (not specified)**	Initial visit with urologist associated with reduced odds of delayed evaluation (OR 0.34, CI 0.31-0.68, p<0.001) compared to primary care and OBGYN (Garg);	Internal medicine providers and other specialists more likely than family physicians to refer (HR 1.30, 1.03-1.64; HR 1.72, 1.01-2.90). No difference in hospital specialists from family medicine (Johnson).	
**Nephrolithiasis**			Delay in non-urologist vs urologist (RR 1.25, CI 1.05-1.49 p=0.01) (Chappidi)
**Benign prostate conditions**			Delay in non-urologist vs urologist (New prostate conditions - RR 1.41, CI 1.12-1.78 p=0.003); recurrent prostate conditions RR 1.94, CI 1.45-2.58 p<0.001) (Chappidi)

* PCP = primary care physician; includes family medicine and internal medicine
+ ED = emergency department
‡ OBGYN = obstetricians and gynaecologists

Figure 1: PRISMA Flow Diagram

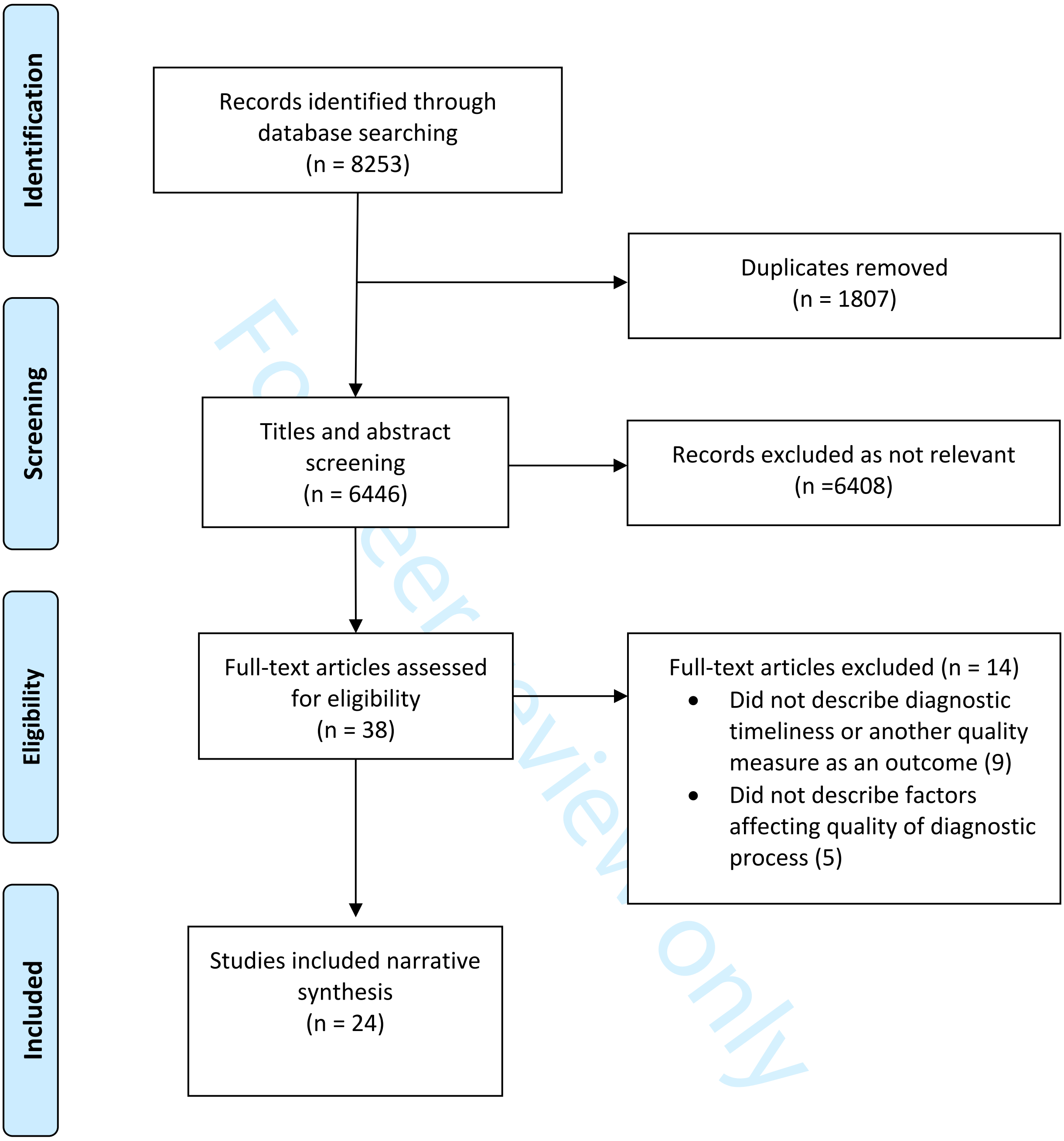

Appendix 1 – Search Strategy

1. exp Urinary Tract Infections/ or exp lower urinary tract symptoms/ or exp hematuria/ or exp polyuria/ or exp nocturia/ or h?ematuri*.ti,ab. Or dysuri*.ti,ab. Or (nocturi* or nycturi*).ti,ab. Or polyuri*.ti,ab. Or exp Urinary Incontinence/ or (urinary adj3 incontinen*).ti,ab. Or (urin* adj3 (frequen* or urgen*)).ti,ab. Or (urin* adj3 infect*).ti,ab. – 379274
2. exp Kidney Neoplasms/ or ((Kidney or renal) adj3 (cancer or neoplas* or tumo?r or carcinoma*)).ti,ab. – 213088
3. exp Urinary Bladder Neoplasms/ or (bladder adj3 (cancer or neoplas* or tumo?r or carcinoma*)).ti,ab. – 145154
4. #2 OR #3 – 346524
5. #1 AND #4 – 20778
6. limit #5 to (humans and yr="2000-Current") – 12950
7. limit #7 to "all adult (19 plus years)" [Limit not valid in Embase; records were retained] – 12268
8. limit #8 to ("all adult (19 plus years)" or "adolescent (13 to 18 years)") [Limit not valid in Embase; records were retained] – 12268
9. limit #6 to (adult <18 to 64 years> or aged <65+ years>) [Limit not valid in Ovid MEDLINE(R),Ovid MEDLINE(R) Daily Update,Ovid MEDLINE(R) In-Process,Ovid MEDLINE(R) Publisher; records were retained] – 8253

For peer review only

Appendix 2: Reported percentages of diagnostic tests and urological referrals for patients with hematuria

Study	Type of hematuria[*]	No evaluation (%)	Frequency of different evaluations and referrals performed (%)							
			History/ Exam	Urine Culture	Urine Cytology	Imaging	Cystoscopy	Both Imaging and cystoscopy	Referral	Guideline concordant evaluation
**Performed within 60 days**										
**Richards 2018**	NVH	81								
**Richards 2018**	VH	67			-					
**Murphy**	VH[+]	47								
**Neider**	NVH			57-60	6-11	20-25			36	
**Performed within 90 days**										
**Richards 2018**	NVH	66								
**Richards 2018**	VH	47								
**Neider**	VH			40-56	5-13	38-41			69-77	
**Performed within 180 days**										
**Bassett**	VH	65						14	23	
**Ark**	NS	54				44	20	18	35	
**Shinagare**	NS	36			43	76	35	64		36[‡]
**Friedlander**	NS					13.9	13.7	5.7		
**Performed within unspecified timescale (1-3 years)**										
**Elias**	NVH	42		15	10	23			21	
**Buteau**	NS	16		63	7.3					
**Bradley (secondary care)**	NVH		63	25						89[§]
**Buteau**	NVH			59	5	34	6	5		
**Buteau**	VH			84	20	42	26	25		

[*]NS: non-specified; NVH: non-visible hematuria; VH: visible hematuria
[+]Murphy included patients with 50 red blood cell per high power field (RBC/HPF), equivalent to very high risk and VH group

For peer review only

Definition of guideline concordant evaluation:
‡ Shinagare: upper renal tract imaging, urine cytology and cystoscopy for high risk patients and 1 urinalysis with >3 RBC/HPF; or upper renal tract imaging followed by either urine cytology or cystoscopy for patients with no risk factors and 2 of 3 urinalysis with >3RBC/HPF.
§ Bradley: cystoscopy and upper tract imaging using CT urography

For peer review only

PRISMA 2009 Checklist

Section/topic	#	Checklist item	Reported on page #
**TITLE**			
Title	1	Identify the report as a systematic review, meta-analysis, or both.	1
**ABSTRACT**			
Structured summary	2	Provide a structured summary including, as applicable: background; objectives; data sources; study eligibility criteria, participants, and interventions; study appraisal and synthesis methods; results; limitations; conclusions and implications of key findings; systematic review registration number.	2-3
**INTRODUCTION**			
Rationale	3	Describe the rationale for the review in the context of what is already known.	4
Objectives	4	Provide an explicit statement of questions being addressed with reference to participants, interventions, comparisons, outcomes, and study design (PICOS).	4-5
**METHODS**			
Protocol and registration	5	Indicate if a review protocol exists, if and where it can be accessed (e.g., Web address), and, if available, provide registration information including registration number.	NA
Eligibility criteria	6	Specify study characteristics (e.g., PICOS, length of follow-up) and report characteristics (e.g., years considered, language, publication status) used as criteria for eligibility, giving rationale.	6-7
Information sources	7	Describe all information sources (e.g., databases with dates of coverage, contact with study authors to identify additional studies) in the search and date last searched.	6
Search	8	Present full electronic search strategy for at least one database, including any limits used, such that it could be repeated.	Appendix 1
Study selection	9	State the process for selecting studies (i.e., screening, eligibility, included in systematic review, and, if applicable, included in the meta-analysis).	6-7
Data collection process	10	Describe method of data extraction from reports (e.g., piloted forms, independently, in duplicate) and any processes for obtaining and confirming data from investigators.	7
Data items	11	List and define all variables for which data were sought (e.g., PICOS, funding sources) and any assumptions and simplifications made.	7
Risk of bias in individual studies	12	Describe methods used for assessing risk of bias of individual studies (including specification of whether this was done at the study or outcome level), and how this information is to be used in any data synthesis.	7
Summary measures	13	State the principal summary measures (e.g., risk ratio, difference in means).	7(narrative)
Synthesis of results	14	Describe the methods of handling data and combining results of studies, if done, including measures of consistency (e.g., I^2) for each meta-analysis.	7

For peer review only

PRISMA 2009 Checklist

Section/topic	#	Checklist item	Reported on page #
Risk of bias across studies	15	Specify any assessment of risk of bias that may affect the cumulative evidence (e.g., publication bias, selective reporting within studies).	7
Additional analyses	16	Describe methods of additional analyses (e.g., sensitivity or subgroup analyses, meta-regression), if done, indicating which were pre-specified.	NA
**RESULTS**			
Study selection	17	Give numbers of studies screened, assessed for eligibility, and included in the review, with reasons for exclusions at each stage, ideally with a flow diagram.	Appendix 1
Study characteristics	18	For each study, present characteristics for which data were extracted (e.g., study size, PICOS, follow-up period) and provide the citations.	Table 1
Risk of bias within studies	19	Present data on risk of bias of each study and, if available, any outcome level assessment (see item 12).	Table 1
Results of individual studies	20	For all outcomes considered (benefits or harms), present, for each study: (a) simple summary data for each intervention group (b) effect estimates and confidence intervals, ideally with a forest plot.	Table 2
Synthesis of results	21	Present results of each meta-analysis done, including confidence intervals and measures of consistency.	NA
Risk of bias across studies	22	Present results of any assessment of risk of bias across studies (see Item 15).	Figure 1
Additional analysis	23	Give results of additional analyses, if done (e.g., sensitivity or subgroup analyses, meta-regression [see Item 16]).	NA
**DISCUSSION**			
Summary of evidence	24	Summarize the main findings including the strength of evidence for each main outcome; consider their relevance to key groups (e.g., healthcare providers, users, and policy makers).	9-15
Limitations	25	Discuss limitations at study and outcome level (e.g., risk of bias), and at review-level (e.g., incomplete retrieval of identified research, reporting bias).	18-19
Conclusions	26	Provide a general interpretation of the results in the context of other evidence, and implications for future research.	15-18
**FUNDING**			
Funding	27	Describe sources of funding for the systematic review and other support (e.g., supply of data); role of funders for the systematic review.	20

From: Moher D, Liberati A, Tetzlaff J, Altman DG, The PRISMA Group (2009). Preferred Reporting Items for Systematic Reviews and Meta-Analyses: The PRISMA Statement. PLoS Med 6(6): e1000097. doi:10.1371/journal.pmed1000097

For more information, visit: **www.prisma-statement.org**.

For peer review only - http://bmjopen.bmj.com/site/about/guidelines.xhtml

BMJ Open

Quality of the Diagnostic Process in Patients Presenting with Symptoms Suggestive of Bladder or Kidney Cancer: A Systematic Review

Journal:	*BMJ Open*
Manuscript ID	bmjopen-2019-029143.R1
Article Type:	Research
Date Submitted by the Author:	27-Jun-2019
Complete List of Authors:	Zhou, Yin; University of Cambridge School of Clinical Medicine, Public Health and Primary Care<br>van Melle, Marije; University of Cambridge Department of Public Health and Primary Care<br>Singh, Hardeep; Michael E. DeBakey Veterans Affairs Medical Center, Houston Veterans Affairs Center for Innovations in Quality, Effectiveness and Safety; Baylor College of Medicine, Department of Medicine<br>Hamilton, Willie; University of Exeter Medical School, Primary Care Diagnostics<br>Lyratzopoulos, Georgios ; University College London, Department of Epidemiology & Public Health, Health Behaviour Research Centre<br>Walter, Fiona; University of Cambridge, Dept of Public Health and Primary Care
**Primary Subject Heading**:	Health services research
Secondary Subject Heading:	Urology, Oncology
Keywords:	Urological tumours < UROLOGY, PRIMARY CARE, Health & safety < HEALTH SERVICES ADMINISTRATION & MANAGEMENT, Quality in health care < HEALTH SERVICES ADMINISTRATION & MANAGEMENT

SCHOLARONE™
Manuscripts

Quality of the Diagnostic Process in Patients Presenting with Symptoms Suggestive of Bladder or Kidney Cancer: A Systematic Review

Yin Zhou, MSc, MRCGP[1] (ykz21@medschl.cam.ac.uk)

Marije van Melle, PhD[1] (mav41@medschl.cam.ac.uk)

Hardeep Singh, MD, MPH[2] (hardeeps@bcm.edu)

Willie Hamilton, MD, FRCGP[3] (w.hamilton@exeter.ac.uk)

Georgios Lyratzopoulos, MD, FFPH, MPH[1,4] (y.lyratzopoulos@ucl.ac.uk)

Fiona M Walter, MD, FRCGP[1] (fmw22@medschl.cam.ac.uk)

[1] The Primary Care Unit, Department of Public Health and Primary Care, University of Cambridge, UK

[2] Center for Innovations in Quality, Effectiveness and Safety, Michael E. DeBakey VA Medical Center, and Department of Medicine, Baylor College of Medicine, Houston, Texas, USA

[3] University of Exeter Medical School, UK

[4] ECHO (Epidemiology of Cancer Healthcare & Outcomes) Research Group, Department of Behavioural Science & Health, University College London, UK

Corresponding author:

Yin Zhou ykz21@medschl.cam.ac.uk +44 (0)1223 748415

Word count: 3701
Abstract: 299
Tables: 4; **Figures:** 1; **Appendices:** 2
References: 49

For peer review only - http://bmjopen.bmj.com/site/about/guidelines.xhtml

ABSTRACT

Objectives

In urological cancers, sex disparity exists for survival, with women doing worse than men. Sub-optimal evaluation of presenting symptoms may contribute.

Design

We performed a systematic review examining factors affecting the quality of the diagnostic process of patients presenting with symptoms of bladder or kidney cancer.

Data Sources

We searched Medline, Embase and the Cochrane Library from 1 January 2000 to 13 June 2019.

Eligible Criteria

We focused on one of the six domains of quality of health care: timeliness, and examined the quality of the diagnostic process more broadly, by assessing whether guideline-concordant history, examination, tests and referrals were performed. Studies describing the factors that affect the timeliness or quality of the assessment of urinary tract infections, haematuria and lower urinary tract symptoms in the context or bladder or kidney cancer, were included.

Data extraction and synthesis

For peer review only - http://bmjopen.bmj.com/site/about/guidelines.xhtml

Data extraction and quality assessment were independently performed by two authors. Due to the heterogeneity of study design and outcomes, the results could not be pooled. A narrative synthesis was performed.

Results:

28 studies met review criteria, representing 583,535 people from 9 high-income countries. Studies were based in primary care (n=8), specialty care (n=12), or both (n=8). Up to two-thirds of patients with haematuria received no further evaluation in the 6 months after their initial visit. Urinary tract infections, nephrolithiasis, and benign prostatic conditions before cancer diagnosis were associated with diagnostic delay. Women were more likely to experience diagnostic delay than men. Patients who first saw a urologist were less likely to experience delayed evaluation and cancer diagnosis.

Conclusions:

Women, and patients with non-cancerous urological diagnoses just prior to their cancer diagnosis, were more likely to experience lower quality diagnostic processes. Risk prediction tools, and improving guideline ambiguity, may improve outcomes and reduce sex disparity in survival for these cancers.

Strengths and Limitations of this study

- This is the first study to our knowledge that examined factors affecting the diagnostic quality of both kidney and bladder cancer.
- We examined all relevant symptoms in these patients, not limiting to haematuria only.

For peer review only - http://bmjopen.bmj.com/site/about/guidelines.xhtml

- We were unable to perform a meta-analysis due to the heterogeneity of the studies.

For peer review only

For peer review only - http://bmjopen.bmj.com/site/about/guidelines.xhtml

INTRODUCTION

Making a correct and timely diagnosis is paramount for patient safety and high quality healthcare. The United States (US) National Academy Sciences, Engineering and Medicine (NASEM- formerly the Institute of Medicine, IOM) report "Improving Diagnosis in Health Care" highlights the importance of research on reducing missed and delayed diagnosis and targeting contributory factors that lead to diagnostic errors[1]. Cancer is one of the commonest conditions to be affected by diagnostic errors[2] and outpatient malpractice claims[3]. This, in addition to the compelling rationale for early detection, makes cancer an excellent disease model for examining diagnostic safety. Bladder and kidney cancer, two relatively common cancers, pose particular diagnostic challenges. Uniquely among common cancers, women with bladder cancer have poorer survival than men with the same cancer[4]. Missed or delayed referral and diagnosis may contribute to the survival difference between men and women with these urological cancers[5].

Timeliness, one of the six domains of health care quality described by the NASEM, can be regarded as the most relevant for evaluating the diagnostic process in cancer[1]. Timely diagnosis of cancer is important to optimise clinical outcomes and patient experience[6,7]. In the United Kingdom (UK), efforts to promote early diagnosis and reducing delays during the diagnostic process have informed many initiatives aiming to improve outcomes for cancer patients[8].

We performed a systematic review to examine the factors affecting the quality of the diagnostic process, in particular timeliness, for patients presenting with urological

For peer review only - http://bmjopen.bmj.com/site/about/guidelines.xhtml

symptoms that may be suggestive of kidney or bladder cancer. Our secondary aim was to examine existing definitions for timeliness of evaluation, referral and diagnosis for these patients.

For peer review only

For peer review only - http://bmjopen.bmj.com/site/about/guidelines.xhtml

METHODS

Search Strategy and Study Inclusion

We searched Ovid Medline and Embase for relevant studies from January 1st 2000 to January 29th 2018, with an updated search on June 13th 2019 of both databases and a new search of the Cochrane Library from inception to the same date. We did not restrict on publication type or languages (Appendix 1). We restricted our search to studies published from 2000 due to prior knowledge that there were few early relevant studies[9], and that the quality of the diagnostic process for cancer might have been affected by the introduction of national initiatives such as the fast-track referral pathways in the United Kingdom (UK) in 2000.

We focused on clinical features listed in the English 2015 National Institute for Health and Care Excellence (NICE) guidelines for suspected cancer[10] in order to examine the population that are most likely to have cancer. We based our outcome measures of diagnostic timeliness on internationally accepted definitions of the diagnostic intervals: e.g. primary care interval = time from the patient's first presentation to a primary care practitioner (PCP), to referral[11,12]. We also examined the quality of the diagnostic process more broadly, by assessing whether appropriate or guideline-concordant history, examination, diagnostic tests and referrals were performed during the evaluation of symptoms.

All titles and abstracts were screened by YZ, with 10% of a random selection independently assessed by a second reviewer (MM). Both authors then independently assessed the full-text

For peer review only - http://bmjopen.bmj.com/site/about/guidelines.xhtml

articles after screening of titles and abstracts. Consensus was sought from GL and FW where disagreements arose.

Inclusion Criteria:

- Studies describing the factors that affect the timeliness or quality of the assessment of the following clinical features in the context or bladder or kidney cancer:
    - Urinary tract infections (UTI)
    - Haematuria
    - Lower urinary tract symptoms (including dysuria, urinary frequency, urgency, incontinence and nocturia)

Exclusion Criteria:

- Studies only describing population or patients under the age of 18
- Conference abstracts, correspondence, editorials, short reports and the grey literature
- Case reports or case series of less than 10 patients.

Data extraction and quality assessment

YZ and MM independently performed data extraction, using a data collection template, on study characteristics, diagnostic intervals, frequency of evaluations, and the patient, clinician and system factors affecting the diagnostic intervals and frequency of evaluations. Quality appraisal was performed using a modified version of the Critical Appraisal Skills Programme (CASP) checklist for cohort studies by both authors (Table 1 Footnote)[13]. Any disagreements were resolved by discussion with all members of the research team.

For peer review only - http://bmjopen.bmj.com/site/about/guidelines.xhtml

Data synthesis and analysis

We were unable to pool the results due to the heterogeneity of the study design and outcomes. A narrative synthesis was therefore performed.

Patient and Public Involvement

No patient was involved in this review.

For peer review only

For peer review only - http://bmjopen.bmj.com/site/about/guidelines.xhtml

RESULTS

Study characteristics and quality

28 papers were included after full-text reviews (Figure 1). All studies were from high-income countries. These include 18 from the US, two from Australia, two from the UK, two from Sweden and one each from Finland, Canada, Austria and Italy, and Germany and Austria (in one study). SIx examined cancer patients with no pre-defined clinical features (five bladder, one both bladder and kidney), five focused on patients with haematuria (one of which included only visible haematuria), eight examined bladder cancer patients with haematuria, and one focused on upper urothelial tract cancer patients with haematuria. Eight studies were carried out in the primary care setting, twelve in hospital and eight in both (Table 1).

The main bias and applicability concerns related to the suboptimal identification and/or adjustment for confounders in 18 of the studies, 6 of which were studies using questionnaires[14-19], 10 were retrospective cohort studies providing descriptive statistics mainly, using record reviews (n=5)[20-24] and electronic health records (n=5)[25-29], 1 was a case-control study[30] and 1 an ecological study[31].

For peer review only - http://bmjopen.bmj.com/site/about/guidelines.xhtml

Table 1: Study characteristics of included publications and quality assessment

Papers	Year/s of data collection	Design	Setting (primary care, specialist or both)	Sample size	Population	CASP Quality Assessment Items								
						A	B	C	D	E	F	G	H	I
Ark 2017, USA	2001-2009	Retrospective cohort study	Primary care	1412	Patients aged 40+ with bladder cancer and haematuria	☺	☺	☺	☺	☺	☺	☺	☺	☺
Aziz 2015, Germany & Austria	2010-2013	Retrospective questionnaire	Specialist	68	Patients undergoing TUR-BT[i] for newly diagnosed urothelial carcinoma of bladder	😐	☹	😐	☹	☹	☹	☹	☹	😐
Bassett 2015, USA	2009-2010	Retrospective cohort study	Primary care	9211	Patients with non-visible haematuria seen by a PCP[ii] in an outpatient setting, age 65+	☺	☺	☺	☺	☺	☺	☺	☺	☺
Blick 2010, UK	1997-2006	Retrospective record review	Specialist	200	Consecutive patients with newly diagnosed bladder cancer identified from the records of MDT[iii] meetings and theatre records	☺	☹	☹	☺	☹	☹	☹	☹	☺
Bradley 2016, USA	2012-2014	Retrospective record review/ cross-sectional cohort	Specialist	237	Women >55 with asymptomatic micro-haematuria	☺	☺	☺	😐	😐	☹	😐	☹	☺
Buteau 2014, USA	2009-2010	Retrospective record review	Primary care	449	Patients with over 5RBC/HPF[iv], with both gross and microscopic haematuria	☺	☺	☺	☺	☺	☹	😐	☹	☺
Chappidi 2017, USA	2010-2014	Retrospective claims review	Both	1326	Patients with UTUC[v] with a haematuria claim 1 year before diagnosis, under 65, 4.4% (n=58) with concomitant bladder cancer diagnosis at time of UTUC diagnosis	☺	☺	☺	☺	☺	☺	☺	☺	☺
Cohn 2014, USA	2004-2010	Retrospective claims review	Both	7649	Patients who had an initial haematuria claim within 12 months of an initial bladder cancer claim, age < 66 (upper limit of MarketScan Database population)	☺	☺	☺	☺	☺	☺	☺	☺	☺
Elias 2010, USA	2006-2007	Retrospective record review	Primary care	164	Participants with microscopic haematuria on urine dipstick testing or 3+RBC/HPF on urinalysis. Recruited from well patient clinics aged 50+ with a 10-year or greater smoking history (any number of cigarettes) and/or a significant (15 or more years) high-risk occupation (such as working in the dye, petroleum, or chemical industry.	☺	☺	☺	☺	☺	☺	☺	☺	☺
Friedlander 2014, USA	2004-2012	Retrospective cohort review	Primary care	2455	Patients aged 40+ with first episode of haematuria between 2004-2012 either by urinalysis (>3RBC/HPF) or ICD diagnosis codes for haematuria	☺	☺	😐	☺	☺	☺	😐	😐	☺
Garg 2014, USA	2000-2007	Retrospective cohort study	Both	35646	Patients aged 66+ with primary bladder cancer with a haematuria claim in 12 months before diagnosis	☺	☺	☺	☺	☺	☺	☺	☺	☺

Han 2018, USA	2014	Cross-sectional, ecological study	Specialist	306 Hospital Referral Regions	Medicare beneficiaries aged 65-99 with bladder cancer and underwent at least one cystoscopy procedure; consisting of 173,551 female and 286,090 men in total	☺	☺	☹	😐	☹	☹	😐	😐	☺
Henning 2013, Austria & Italy	Not mentioned	Prospective questionnaire study	Specialist	200	Consecutive series of 200 patients admitted for elective TUR-BT	☺	☺	☹	😐	☹	☹	😐	😐	☺
Hollenbeck 2010, USA	1992-2002	Retrospective cohort study	Both	29740	Patients with haematuria 1 year prior to bladder cancer diagnosis; 66+	☺	☺	☺	☺	☺	☹	😐	😐	☺
Johnson 2008, USA	1998-2002	Retrospective cohort study	Both	926	Patients with newly diagnosed haematuria, 18+	☺	😐	☺	😐	😐	😐	☺	☺	☺
Liedberg 2016, Sweden	2014-2015	Interventional study	Both	275	Patients in intervention group, 101 in control group; patients=50+ with macroscopic haematuria	☺	😐	😐	☺	☹	☹	😐	😐	☺
Lyratzopoulos 2013, UK	2009-2010	Secondary analysis of audit data	Primary care	1318	Bladder and kidney cancer patients	☺	☺	☺	☺	☺	☺	☺	☺	☺
Matulewicz 2019, USA	2007-2015	Retrospective cohort study	Multi-institutional hospital system	15,161	Microscopic haematuria in patients aged 35 and over	☺	☺	☺	☺	☺	😐	☺	☺	☺
McCombie 2017, Australia	2008-2014	Retrospective cohort study, telephone survey	Specialist	100	Bladder cancer patients	☺	☺	😐	☺	☹	☹	😐	☹	☺
Murphy 2017, USA	2012-2014	Retrospective cohort study	Primary care	495	Patients with haematuria (urine red blood cells of >50 cells per high power field)	☺	😐	😐	☺	☹	☹	😐	😐	☺
Ngo 2017, Australia	2015 (12 month period)	Retrospective record review	Specialist	305	Cystoscopy cases primarily for haematuria investigation, 18+	☺	☺	☺	☺	☺	☺	☺	😐	☺
Nieder 2010, USA	Not mentioned	Questionnaire study	Primary care	788	788 PCPs (internal medicine, family practice, primary care, or obstetrics and gynaecology) randomly selected from the Little Blue Book of Miami-Dade County and Dallas, published by National Physicians Data Source	☺	☺	😐	☹	☹	☹	😐	😐	☺
Richards 2016, USA	2007-2009	Retrospective cohort study	Both	12195	Patients from SEER-Medicare with a haematuria or UTI[vi] claim; 66+	☺	☺	☺	☺	☺	☺	☺	☺	☺

						A	B	C	D	E	F	G	H	I
Nilbert 2018, Sweden	2015-2016	Case control study	Secondary care	1,697 controls / 174 cases	Patients with macroscopic haematuria (control) and bladder and upper urothelial tract cancer (cases), aged 40 and over	☺	☺	😐	☺	😐	☹	☺	☺	☺
Richards 2018, USA	2011-2013	Retrospective record review	Both	201	Consecutive patients with new-onset haematuria, 195 male	☺	😐	☺	☺	😐	☺	☺	😐	☺
Santos 2015, Canada	2000-2009	Retrospective cohort review	Specialist	1271	Patients who underwent radical cystectomy for bladder cancer, had a first urologist visit after having visited a GP[vii] or ED[viii] physician, >40+ age	☺	😐	😐	😐	😐	☹	😐	☹	☺
Sell 2019, Finland	Unknown	Sub-study of RCT[ix], using questionnaires	Specialist	131	Patients with histologically proven low-grade bladder cancer with self-reported macroscopic haematuria	☺	☺	😐	😐	☹	☹	😐	☺	☺
Shinagare 2014, USA	2004-2012	Retrospective record review	Specialist	100	Consecutive patients with asymptomatic haematuria	☺	😐	☺	☺	☹	😐	😐	☹	😐

Footnotes

[i] TUR-BT: transurethral resection of bladder tumour
[ii] PCP: primary care practitioner
[iii] MDT: multidisciplinary team
[iv] RBC/HPF: red blood cell per high power field
[v] UTUC: upper tract urothelial carcinoma
[vi] UTI: urinary tract infection
[vii] GP: general practitioner
[viii] ED: emergency department
[ix] RCT: randomised controlled trial

CASP Quality Assessment Items (Key: green=yes, yellow=can't tell, red=no)
A: Does the study address a clearly focused issue?
B: Was the cohort recruited in an acceptable way?
C: Was the exposure accurately measured to minimise bias?
D: Was the outcome accurately measured to minimise bias?
E: Have the authors identified all important confounding factors?
F: Have they taken account of the confounding factors in the design and/or analysis?
G: Do you believe the results?
H: Can the results be applied to the local population?
I: Do the results of this study fit with the other available evidence?

For peer review only - http://bmjopen.bmj.com/site/about/guidelines.xhtml

Quality of Diagnostic Process

Diagnostic timeliness

Seventeen of the 28 included studies described diagnostic intervals for patients with either urological symptoms or who had been diagnosed with bladder or kidney cancer (Table 2).

Definitions of timely evaluation, referral and diagnosis, were described in 12 studies. For time to first evaluation including cystoscopy, upper urinary tract imaging or urine cytology, Garg et al. used a threshold of 30 days[32], while two studies examined proportions of patients undergoing these tests within 60[33] and 90 days[24]. The remaining studies used 180 days as time cut-offs for which they considered evaluation should be carried out[22,28,34-36], although one also looked at completion within 365 years, and beyond[28]. Thresholds for referral was set at 90 days by one paper[27], while delays in cancer diagnosis were defined as greater than 90 days[37] and in 3 month increments up to 1 year[26,38].

For peer review only

Table 2: Mean and median diagnostic intervals from first presentation to diagnosis as described in included studies

Study	Population of interest^	Time interval ‡	Event points (shading denotes interval): First presentation with symptom	Referral to specialist	First specialist appointment	First investigation	Diagnosis	Mean interval duration (days): All	Men	Women	Median interval duration (days): All	Men	Women
**Aziz 2015, Austria/ Germany**	VH[+]	T1						335	343.70	313.29			
**Chappidi 2017, USA**	UTUC[+] with haematuria	T1							93.5*	84.4*		60*	49*
**Cohn 2014, USA**	Bladder	T1							85.4*	73.6*		41*	35*

	cancer with haematuria								
**Liedberg 2016, Sweden**	VH^{+}	T1					29 vs 50*	(intervention vs control)	
**Nilberg 2018, Sweden**	Bladder/ UTUC with VH	T1					25 vs 35*	(intervention vs control)	
**Richards 2016, USA**	Bladder	T1			58.9*	72.2*			
**Garg 2014, USA**	Bladder cancer with haematuria	T2					8	8	9
**Matulewicz 2019, USA**	VH	T2	Time to imaging				75		
**Matulewicz 2019, USA**	VH	T2	Time to cystoscopy				68.5		
**Garg 2014, USA**	Bladder cancer with haematuria	T3		27	24*	35*	3	2*	6*
**Richards 2018, USA**	NVH^{+}	T3					28		
**Santos 2015, USA**	Bladder	T3					30	23*	56*
**Chappidi 2017, USA**	UTUC^{+} with haematuria	T3			17.9	20.4		4	5
**Johnson 2008, USA**	Haematuria	T4					33.5	27.4 *	36.5*
**Liedberg 2016, Sweden**	VH^{+}	T4					14 vs 33	(intervention vs control)	
**Lyratzopoulos 2013, UK**	Bladder	T4						4*	6*
**Lyratzopoulos 2013, UK**	Kidney	T4						10*	16*
**McCombie 2017, Australia**	Bladder	T4					3		
**Nilberg 2018, Sweden**	Bladder/ UTUC with VH	T4					0 vs 7	(intervention vs control)	
**Sell 2019, Finland**	Bladder with VH	T4					8		
**Sell 2019, Finland**	Bladder with VH	T6+T7	Urology referral to cystoscopy				23		
**Blick 2010, UK**	Bladder	T6		21.3					
**McCombie 2017, Australia**		T6					23.5		
**McCombie 2017, Australia**		T6a					3		
**McCombie 2017, Australia**		T6b					19.5		
**Ngo 2017 Australia**	Haematuria	T6					38		
**Ngo 2017 Australia**	Haematuria	T7					28		

^ Population of interest indicates the characteristics of the cohort examined in each study (either symptom or type of cancer patients)
* p-value ≤ 0.05 for difference between men and women
$^{+}$ VH = visible haematuria; NVH = non-visible haematuria; UTUC: upper tract urothelial carcinoma
‡ Key for time intervals:
T1: Time from first presentation with symptom to diagnosis
T2: Time from first presentation with symptom to first investigation
T3: Time from first presentation to first specialist appointment
T4: Time from first presentation to referral to specialist
T5: Time from referral to specialist to diagnosis

For peer review only - http://bmjopen.bmj.com/site/about/guidelines.xhtml

T6: Time from referral to specialist to first specialist appointment
T6a: Time from referral being sent to referral being received
T6b: Time from receipt of referral to first specialist appointment
T7: Time from first specialist appointment to first investigation

Other Quality Dimensions

We found no standard definition for high quality care during the diagnostic process in any of the included studies. Most studies reported the frequency of appropriate or guideline-concordant diagnostic tests and referrals performed during diagnostic evaluation (Appendix 2). Studies examining the frequency of non-evaluation of haematuria reported this to be 47 – 81% within 60 days of initial symptom presentation[24,33], reducing to 36 – 65 % in studies by 180 days[22,34-36].

Eleven studies reported the percentages of investigations and referrals performed in patients with haematuria[18,21,22,24,25,28,33-36,39], seven of which also specified time-frames during which these evaluations should be completed[22,24,28,33-36] (Appendix 2). Five studies reported that only 5 – 25% of these patients received both imaging and cystoscopy (commonly defined as 'complete evaluation' by the studies) by 6 months of their first presentation with haematuria[25,28,34-36] and case series of 100 patients in a single institution reported this percentage to be 64% in their cohort[22].

Studies reported variations in the percentages of patients with haematuria who received urine culture (15 – 84%), urine cytology (5 – 43%), imaging tests (14 – 76%), and cystoscopy (6 – 26%) at at least 2 months after presentation, indicating that there were variations in how clinicians evaluate patients with haematuria. In studies that focused on the type of haematuria, a larger proportion of patients with visible haematuria (VH) (25%)[25] received

For peer review only - http://bmjopen.bmj.com/site/about/guidelines.xhtml

both imaging and cystoscopy than patients with non-visible haematuria (NVH) (up to 14%)[25,28,35]. Between 21-36% of patients with haematuria received a urological referral[34,35,39], although a survey study from almost 800 PCPs in the US reported that about a third and two-thirds of them would refer patients with NVH and VH respectively[18].

One secondary care study reported non guideline-compliant practice in over a third of post-menopausal women with asymptomatic haematuria, with no documentation of full genitourinary examination with vaginal tissue quality and presence of prolapse[21].

Presenting symptoms

Ten of the 20 studies which extracted symptom information used coded information from routine or claims data[21,25,28,29,31,34,35,37-39], five used self-reported symptoms from questionnaire and audit data[14-16,19,40], six studies performed direct record review[22,24,25,30,33,41], and one used coded information and record review[17].

Eleven studies examined the direct association between the type of presenting symptoms and quality of the diagnostic process, the majority focusing on haematuria[18,19,22,24,27,28,30,38,40,41] and one on UTI [42]. No other presenting symptoms were examined. A large population-based study in the UK reported that bladder or kidney cancer patients presenting with haematuria were significantly less likely to have 3 or more consultations before a GP referral compared to those who did not present with haematuria (OR 0.29 CI 0.19-0.46, p<0.001 for bladder cancer; OR 0.64 CI 0.30-1.37, p=0.25 for kidney cancer)[40]. An earlier study of about 1000 patients found that there was also a dose-dependent relationship between the number of haematuria visits and the likelihood of a

For peer review only - http://bmjopen.bmj.com/site/about/guidelines.xhtml

urological referral (HR 5.18 and 7.66 for 2 and 3 visits respectively vs 1 visit, p<0.0001)[27], and a high-quality US study involving claims review found that increasing number of haematuria visits was associated with diagnostic delay for bladder cancer patients[38].

VH predicted a shorter time to evaluation[38], to referral[19], a lower likelihood of incomplete evaluation[24 22], shorter time from GP referral to urology consultation[41], and a shorter time to diagnosis[38] than NVH.

Other recent diagnoses preceding cancer diagnosis

Between 20-61% of women and 15-35% of symptomatic men were treated or diagnosed with a UTI before being diagnosed with bladder cancer[14,15,37,38]. Women are also four times as likely as men to receive three or more courses of treatments for UTIs before their cancer diagnosis (15.8% vs 3.8% for women vs men, p=0.04)[15].

In two case series it was also reported that a significant proportion of bladder cancer patients (up to 40%) received symptomatic treatments for either lower urinary tract symptoms or abdominal pain before referral to a urologist[14] or were not further evaluated, with women more likely to be affected than men (41.7% vs 16.2% once or twice, 5.6% vs 2.9% three or more times, women vs men; p=0.04)[15].

Two large US studies reported that benign diagnosis (up to 12 months prior to cancer diagnosis) such as UTIs, nephrolithiasis and prostate-related diagnosis were associated with delays in cancer diagnosis[37,38]. UTIs were associated with a two-fold increase in the odds of

For peer review only - http://bmjopen.bmj.com/site/about/guidelines.xhtml

diagnostic delay by at least 3 months in both sexes for both upper tract urothelial cancer and bladder cancer[37,38], compared to those with no UTI diagnosis pre-diagnosis (OR 1.97, CI 1.74-2.22)[38]. This was regardless of whether patients first presented to a urologist or other specialty doctors[37]. Nephrolithiasis (RR 1.29, CI 1.07-1.54; p=0.007 vs RR 1.09, 0.81-1.47 for men vs women) and benign prostatic conditions (such as prostatitis, benign prostatic hyperplasia and benign prostatic nodule) were more likely to predict diagnostic delay in men than women with upper tract urothelial cancer[37]. In this nationwide insurance claims study, no validation of coded information was performed using medical record review.

Patient factors

Sex

The effect of sex on diagnostic activity and timeliness were reported by 15 studies[14,15,19,22,25,27-29,31,32,34,36,38-40]. Most evidence indicated that women were less likely than men to undergo any investigation[35], have complete evaluation[28,35], be referred to a specialist [27,29,41], and to have cystoscopy or imaging[31,36,41]. Female sex was also a consistent predictor for delayed evaluation of haematuria[32,34,38] and UTIs[15,25], and longer diagnostic intervals for cancer[29,32,40]. This sex disparity with respect to evaluation and referral was insignificant for patients with NVH[22,25,39]. One Finnish study using patient questionnaires reported no difference in patient (time from symptom recognition to presentation) and primary care (time from presentation to referral to a urologist) intervals in 131 low-grade bladder cancer patients between men and women[19].

Other patient factors

For peer review only - http://bmjopen.bmj.com/site/about/guidelines.xhtml

In general, evidence for other patient factors affecting the quality of the diagnostic process was less consistent. Two large US studies consisting of over 65,000 patients in total found that older bladder cancer patients with haematuria had longer delays to evaluation than younger patients[26,32]. Increasing comorbidity predicted slower time to urologist, longer delay to evaluation[32], and diagnosis[38] in two large US samples. While five of the six studies reported no association between ethnicity and quality of the diagnostic process, one study with about 1400 participants reported that African-American bladder cancer patients were less likely than their Caucasian counterparts to: be referred to a urologist (adjusted OR 0.72; 95% CI 0.56-0.93; have a cystoscopy (adjusted OR 0.67; 95% CI 0.50-0.89), or have imaging (adjusted OR 0.75; 95% CI 0.59-0.95)[34].

The evidence between socioeconomic status, comorbidity, smoking, and anticoagulant use was either non-significant or weak (Table 3).

Table 3: Summary of association between patient factors and diagnostic safety and timeliness

Patient factor	Number of studies exploring risk factor	Association between patient factor and diagnostic safety and timeliness		
		Delayed / Incomplete Evaluation	Delayed Referral	Longer Diagnostic interval
Sex	15	Women>Men	Women>Men	Women >Men
Increasing age	12	NS [24,25,28,39]<br>Positive association [26,32]	NS [19,25,32,41]	NS [38]
Ethnicity	7	NS [25,26,28,32,35,36]<br>African-American worse [34]	NS [25,26,32,35,36]<br>African-American worse [34]	NS [25,26,32,35,36]
SES	5	NS [16,26,36,38,41]		
Comorbidity	4	Positive association [32]<br>NS [24,37]		Positive association [38]<br>NS [24,37]
Smoking	6	NS [25,35,36,41]<br>Positive association [39]	NS [19,25,35,36,41]	
Anticoagulant use	5	NS [24,35,41]<br>More likely to receive imaging [36]	NS [24,30,35,41]	NS [30]

For peer review only - http://bmjopen.bmj.com/site/about/guidelines.xhtml

NS: statistically non-significant

Clinician factors

Physician type

Six studies from the US examined the type of clinicians as a predictor for diagnostic delay (Table 4). Patients who first saw a urologist for their symptoms were less likely to have a delay in evaluation[32] or cancer diagnosis[37] and more likely to have guideline-adherent evaluation[22] than those who first saw another specialty doctor.

When comparing specialties excluding urology, a mixed pattern was seen with respect to referral and use of investigations. In general, there was little evidence to suggest that family physicians in the US differ from other specialists with respect to evaluating haematuria. Family physicians may be less likely to refer for VH[25,27] although evidence for delayed referral in patients with UTI and NVH was less clear[18,25].

Table 4: Associations between physician specialty and quality of diagnostic process for patients presenting with different clinical features pre-cancer diagnosis

Clinical feature	Delay in evaluation	Delay in referral	Delay in diagnosis
**Urinary tract infection (UTI)**	No difference between PCP* and other specialists or ED+ physicians (Buteau)	No difference between PCP* and other specialists or ED+ physicians (Buteau)	Both urologist and non-urologist (Urologist: RR1.74, CI 1.31-2.31 p<0.001; non-urologist RR 1.44, CI 1.22-1.71 p<0.001 (Chappidi)

For peer review only - http://bmjopen.bmj.com/site/about/guidelines.xhtml

**Microscopic haematuria**	No difference between PCP[*] and other specialists or ED[+] physicians (Buteau); OBGYN[‡] less likely to perform imaging than medical counterparts (p<0.004) (Neider); guideline concordant with urologist vs non-urologist (OR 54.7, CI 10-102, p<0.0001) (Shinagare)	No difference between PCP[*] and other specialists or ED[+] physicians (Buteau, Neider)	
**Macroscopic haematuria**	OBGYN[‡] less likely to perform imaging than medical counterparts (p<0.01) (Neider)	PCP[*] less than other specialists or ED+ physicians (Buteau); no difference between specialties (Neider)	
**Haematuria (not specified)**	Initial visit with urologist associated with reduced odds of delayed evaluation (OR 0.34, CI 0.31-0.68, p<0.001) compared to primary care and OBGYN (Garg);	Internal medicine providers and other specialists more likely than family physicians to refer (HR 1.30, 1.03-1.64; HR 1.72, 1.01-2.90). No difference in hospital specialists from family medicine (Johnson).	
**Nephrolithiasis**			Delay in non-urologist vs urologist (RR 1.25, CI 1.05-1.49 p=0.01) (Chappidi)
**Benign prostate conditions**			Delay in non-urologist vs urologist (New prostate conditions - RR 1.41, CI 1.12-1.78 p=0.003); recurrent prostate conditions RR 1.94, CI 1.45-2.58 p<0.001) (Chappidi)

[*] PCP = primary care physician; includes family medicine and internal medicine
[+] ED = emergency department
[‡] OBGYN = obstetricians and gynaecologists

System factors

Diagnostic pathways

Three studies examined the impact of interventions in the diagnostic pathways on diagnostic intervals in the UK and Sweden[16,20,30]. A single institution UK study found that the time from general practitioner (GP) referral to first hospital visit shortened from 42.9 days to 21.3 days (p<0.001) after the introduction of the fast-track pathway, in which patients with alarm symptoms are typically seen or investigated by a specialist within two weeks of a

For peer review only - http://bmjopen.bmj.com/site/about/guidelines.xhtml

GP referral[20]. In Sweden, the introduction of a telephone hotline for patients with VH reduced the time from haematuria to urology referral (33 to 14 days, p=0.32), referral to diagnosis (19 to 8 days, p=0.003), and total healthcare interval (50 to 29 days, p=0.03)[16]. Patients with eligible symptoms were able to access a nurse consultant directly by telephone, who then scheduled the patient for serum creatinine, urine cytology and appointment with a urologist for flexible cystoscopy and CT urography within 2 weeks, all with the same priority as other patients referred by their GP but bypassing the routine referral system[16]. Another Swedish study studying a similar streamlined diagnostic pathway found that it shortened the diagnostic interval from 35 to 25 days (p=0.01) although time to treatment did not change from a regular referral pathway[30].

Other factors

Other factors that were found to impact on the quality of the diagnostic process were described by studies using direct record review. These include patient factors such as not attending, cancelling or declining to attend follow up appointments[33], delays in PCPs reviewing results, lack of receipt of referral[17], and scheduling and coordination delay of follow-up test or appointment[24,33], although the detailed effects of these factors on the quality of the diagnostic process were not reported.

DISCUSSION

Our review identified several potential areas of missed opportunities in urological cancer diagnosis; it also provides evidence for informing the development of future interventions and research.

For peer review only - http://bmjopen.bmj.com/site/about/guidelines.xhtml

Non-evaluation of haematuria

Studies reported high frequencies of non-evaluation of haematuria, with about two-thirds of patients having no evaluation up to 180 days after initial presentation. Although we found no consistent definition of diagnostic timeliness for evaluation, referral and diagnosis of urological cancer, high percentages of non-evaluated cases likely harbor missed opportunities for a timely diagnosis. For instance, patients with VH should receive renal function testing, imaging and urology referral for cystoscopy once a transient cause such as UTI has been excluded[43]. Patients with persistent NVH should additionally receive a blood pressure check and urinary albumin-creatinine ratio as part of the evaluation[43]. Given that the PPV of haematuria for urological cancer can be as high as 11%[44], lack of evaluation could lead to missed diagnoses. Improving clinicians' awareness and adherence to existing guidelines as well as using electronic algorithms to flag up abnormal findings[33] may reduce such missed opportunities[23].

Women experience poorer quality of diagnostic process than men

Our review found that women with haematuria were more likely to be treated for UTIs or for pain, and less likely to be evaluated further or referred than men. Benign conditions such as UTIs and atrophic vaginitis are commoner in women. At the same time women are less likely to have bladder and kidney cancer than men (M: F ratio about 3:1 and 1.7:1 for bladder and kidney respectively)[45]. This may be due to differing exposure to lifestyle and environmental factors, and biological propensity to these cancers by sex[4]. The combined effect of greater frequency of non-neoplastic disease in women, and the fact that urological

For peer review only - http://bmjopen.bmj.com/site/about/guidelines.xhtml

cancer is less common in women, means that the PPV of relevant symptoms for bladder and kidney cancer is lower in women than men.

Although a relevant urological symptom is more likely to be due to a benign cause in women than in men, avoidable diagnostic delay for urological cancer may still occur if there is a failure to ensure the resolution of symptoms. Current American Urological Association (AUA) guidelines on the evaluation of asymptomatic microscopic haematuria recommends repeat urinalysis after the treatment of other causes, and subsequent renal function testing, cystoscopy and imaging if symptoms do not resolve after treatment[46]. However, the low diagnostic yield of NVH evaluation and the frequency of other benign causes (such as infection) in everyday clinical practice may affect guideline adherence, and contribute to diagnostic delay. Future research should examine risk stratification based not only on presence of symptoms, but also on their severity, chronicity, recurrence or persistent nature. For example, guidelines should address cut-offs for degree of NVH that should warrant active reviews after treatments for UTIs; or cut-offs for number of UTIs treated before referral), while also taking into account cost-effectiveness of any follow-up actions. Emerging urine biomarkers and risk prediction tools may also be useful additions to improve diagnostic yield[47,48].

Concomitant benign conditions

The challenge in clinical practice arises when a presenting symptom may be the result of concomitant benign disease or cancer. In these cases it is likely that a significant proportion of patients will be appropriately treated and reviewed, averting unnecessary investigations.

For peer review only - http://bmjopen.bmj.com/site/about/guidelines.xhtml

The observation that a UTI diagnosis delays cancer diagnosis in patients first seen by both urologists and non-urologists[37] suggests that diagnostic reasoning is challenging for clinicians in such situations. The PPV of a symptom for cancer probably falls if the patient has concomitant diseases which cause the symptom. Some evidence suggests that conditions such as benign prostate disease and kidney stones also delay the diagnosis of kidney cancer [37]. Whether and how much of this diagnostic delay is avoidable is yet to be determined, and should be a priority for future research. While current UK guidelines recommend that patients with persistent or recurrent UTIs should be referred for further evaluation, there is no US equivalent, nor guidelines on the management of other concomitant benign conditions and possible cancer. When no guideline exists, or adherence is not possible, close follow-up to ensure improvement or resolution of symptoms should take place, and patients should be instructed to return if symptoms do not improve.

Clinician and system factors

Patients experienced shorter diagnostic intervals if they first presented to a urologist instead of another specialty doctor. This is not surprising given that urologists are likely to have better access to investigations, typically consisting of cystoscopy and upper renal tract imaging. The variations seen in evaluation and referral between other clinical specialties may indicate different levels of guideline awareness and adherence, although this evidence is scarce and inconsistent.

Process or system delays, such as patient non-attendance at appointments, delays in scheduling of appointments or non-receipt of referrals, all contribute to diagnostic delay[17,24,33,49], although the magnitude of their effects is unclear. In addition to improving

For peer review only - http://bmjopen.bmj.com/site/about/guidelines.xhtml

process and workflow issues within primary and secondary care services, wider system changes such as providing direct access to imaging and streamlining referral processes may also play a role in expediting cancer diagnosis. Diagnostic pathways such as the fast-track referral system for patients with alarm symptoms in the UK, or a telephone hotline service, may shorten primary care and total healthcare interval, although the cost-effectiveness of such pathways need to be evaluated in specific health system contexts.

Strengths and limitations

Our review is the first to examine the evidence relating to factors affecting the quality of the diagnostic process in patients with bladder and kidney cancer. It builds on a previous review examining haematuria assessment in bladder cancer patients[9], and looks at a range of urological symptoms, and also patients with kidney cancer. Although some of the studies did not adjust for all the confounders, the descriptive sections related mainly to the diagnostic intervals and appropriate statistical analyses were performed for examining the factors affecting these intervals, where relevant.

Unfortunately, we were unable to perform a meta-analysis due to the heterogeneity in study designs and outcomes. We were also unable to check the veracity of the co-morbid disease labels in the papers that used coded information. All the studies are from high-income countries, and therefore may be less generalizable to other countries with differing health care systems.

Conclusion

For peer review only - http://bmjopen.bmj.com/site/about/guidelines.xhtml

We found lack of consistency in defining diagnostic quality, including timeliness, of bladder and kidney cancer, and insufficient exploration of population-based evidence related to clinician and system factors affecting the quality of the diagnostic process. Our review highlights the need to improve evaluation of haematuria, and to develop high-quality evidence to inform guidelines on specific thresholds for active follow-up of high-risk symptomatic patients, which could be incorporated into risk prediction tools and clinical decision support. Future research should also identify and target preventable delays, especially in the context of concomitant benign conditions. Identifying patients with evaluation delays through electronic algorithms may also improve outcomes and reduce the sex inequality in survival for these cancers. In sum, our review identifies several potential areas of missed opportunities in bladder and kidney cancer diagnosis that may be avoidable and amenable to targeted interventions.

Figure 1: PRISMA Flow Diagram

For peer review only - http://bmjopen.bmj.com/site/about/guidelines.xhtml

Acknowledgements

The authors would like to thank Isla Kuhn, medical librarian at the University of Cambridge School of Clinical Medicine Medical Library, for her advice and assistance with the development of the strategy strategy.

Funding

YZ is supported by a Wellcome Trust Primary Care Clinician PhD Fellowship (203921/Z/16/Z). The authors WH and FW are co-Principal Investigators and the authors YL and HS are co-Investigators of the multi-institutional CanTest Research Collaborative funded by a Cancer Research UK Population Research Catalyst award (C8640/A23385). HS is additionally supported by the Houston VA HSR&D Center for Innovations in Quality, Effectiveness and Safety (CIN 13-413).

Declaration of Interest

All authors declare no conflicts of interest.

Data availability

All data relevant to the study are included in the article or uploaded as supplementary information.

Author contribution

YZ, GL and FW designed the study. YZ developed and performed the search. YZ and MM performed the data extraction with MM. YZ drafted the manuscript, MM, HS, WH, GL and FW critically revised the article.

For peer review only - http://bmjopen.bmj.com/site/about/guidelines.xhtml

References

1. Institute of Medicine. Improving diagnosis in health care. 2015; https://www.nap.edu/catalog/21794/improving-diagnosis-in-health-care. Accessed August 26th 2018.
2. Kostopoulou O, Delaney BC, Munro CW. Diagnostic difficulty and error in primary care—a systematic review. *Family practice.* 2008;25(6):400-413.
3. Gandhi TK, Kachalia A, Thomas EJ, et al. Missed and delayed diagnoses in the ambulatory setting: a study of closed malpractice claims. *Annals of internal medicine.* 2006;145(7):488-496.
4. Dobruch J, Daneshmand S, Fisch M, et al. Gender and bladder cancer: a collaborative review of etiology, biology, and outcomes. *European urology.* 2016;69(2):300-310.
5. Neal R, Tharmanathan P, France B, et al. Is increased time to diagnosis and treatment in symptomatic cancer associated with poorer outomes? Systematic review. *British journal of cancer.* 2015;112:S92-S107.
6. Mendonca SC, Abel GA, Saunders CL, Wardle J, Lyratzopoulos G. Pre‐referral general practitioner consultations and subsequent experience of cancer care: evidence from the English Cancer Patient Experience Survey. *European Journal of Cancer Care.* 2015;25(3):478-490.
7. Hamilton W, Walter FM, Rubin G, Neal RD. Improving early diagnosis of symptomatic cancer. *Nature Reviews Clinical Oncology.* 2016;13(12):740-749.
8. Hiom S. Diagnosing cancer earlier: reviewing the evidence for improving cancer survival. *British journal of cancer.* 2015;112:S1-S5.
9. Ngo B, Perera M, Papa N, Bolton D, Sengupta S. Factors affecting the timeliness and adequacy of haematuria assessment in bladder cancer: a systematic review. *BJU International.* 2017;119 Suppl 5:10-18.
10. NICE. NICE guidelines [NG12]: Suspected cancer: recognition and referral. 2015; http://www.nice.org.uk/guidance/NG12/. Accessed 1st September 2018.
11. Walter F, Webster A, Scott S, Emery J. The Andersen Model of Total Patient Delay: a systematic review of its application in cancer diagnosis. *Journal of health services research & policy.* 2012;17(2):110-118.
12. Weller D, Vedsted P, Rubin G, et al. The Aarhus statement: improving design and reporting of studies on early cancer diagnosis. *British Journal of Cancer.* 2012;106(7):1262-1267.
13. Critical Appraisal Skills Programme. CASP Cohort Study Checklist. 2017; http://www.casp-uk.net/checklists. Accessed 1st September 2018.
14. Aziz A, Madersbacher S, Otto W, et al. Comparative analysis of gender-related differences in symptoms and referral patterns prior to initial diagnosis of urothelial carcinoma of the bladder: a prospective cohort study. *Urologia Internationalis.* 2015;94(1):37-44.
15. Henning A, Wehrberger M, Madersbacher S, et al. Do differences in clinical symptoms and referral patterns contribute to the gender gap in bladder cancer? *BJU International.* 2013;112(1):68-73.
16. Liedberg F, Gerdtham U, Gralen K, et al. Fast-track access to urologic care for patients with macroscopic haematuria is efficient and cost-effective: results from a prospective intervention study. *British Journal of Cancer.* 2016;115(7):770-775.

For peer review only - http://bmjopen.bmj.com/site/about/guidelines.xhtml

17. McCombie SP, Bangash HK, Kuan M, Thyer I, Lee F, Hayne D. Delays in the diagnosis and initial treatment of bladder cancer in Western Australia. *BJU International.* 2017;120 Suppl 3:28-34.
18. Nieder AM, Lotan Y, Nuss GR, et al. Are patients with hematuria appropriately referred to Urology? A multi-institutional questionnaire based survey. *Urologic Oncology.* 2010;28(5):500-503.
19. Sell V, Ettala O, Montoya Perez I, et al. Symptoms and diagnostic delays in bladder cancer with high risk of recurrence: results from a prospective FinnBladder 9 trial. *World Journal of Urology.* 2019:08.
20. Blick C, Bailey D, Haldar N, Bdesha A, Kelleher J, Muneer A. The impact of the two-week wait rule on the diagnosis and management of bladder cancer in a single UK institution. *Annals of the Royal College of Surgeons of England.* 2010;92(1):46-50.
21. Bradley MS, Willis-Gray MG, Amundsen CL, Siddiqui NY. Microhematuria in Postmenopausal Women: Adherence to Guidelines in a Tertiary Care Setting. *Journal of Urology.* 2016;195(4 Pt 1):937-941.
22. Shinagare AB, Silverman SG, Gershanik EF, Chang SL, Khorasani R. Evaluating hematuria: Impact of guideline adherence on urologic cancer diagnosis. *American Journal of Medicine.* 2014;127(7):625-632.
23. Murphy DR, Wu L, Thomas EJ, Forjuoh SN, Meyer AN, Singh H. Electronic trigger-based intervention to reduce delays in diagnostic evaluation for cancer: a cluster randomized controlled trial. *Journal of Clinical Oncology.* 2015;33(31):3560.
24. Richards KA, Ruiz VL, Murphy DR, et al. Diagnostic evaluation of patients presenting with hematuria: An electronic health record-based study. *Urologic Oncology: Seminars and Original Investigations.* 2018;36(3):88.e19-88.e25.
25. Buteau A, Seideman CA, Svatek RS, et al. What is evaluation of hematuria by primary care physicians? Use of electronic medical records to assess practice patterns with intermediate follow-up. *Urologic Oncology: Seminars and Original Investigations.* 2014;32(2):128-134.
26. Hollenbeck BK, Dunn RL, Ye Z, et al. Delays in diagnosis and bladder cancer mortality. *Cancer.* 2010;116(22):5235-5242.
27. Johnson EK, Daignault S, Zhang Y, Lee CT. Patterns of hematuria referral to urologists: does a gender disparity exist? *Urology.* 2008;72(3):498-502; discussion 502-493.
28. Matulewicz RS, Demzik AL, DeLancey JO, Popescu O, Makarov DV, Meeks JJ. Disparities in the diagnostic evaluation of microhematuriaand implications for the detection of urologic malignancy. *Urologic Oncology: Seminars and Original Investigations.* 2019;37(5):300.e301-300.e307.
29. Santos F, Dragomir A, Kassouf W, Franco E, Aprikian A. Urologist referral delay and its impact on survival after radical cystectomy for bladder cancer. *Current Oncology.* 2015;22(1):e20-e26.
30. Nilbert M, Blackberg M, Ceberg J, Hagberg O, Stenhoff R, Liedberg F. Diagnostic pathway efficacy for urinary tract cancer: population-based outcome of standardized evaluation for macroscopic haematuria. *Scandinavian Journal of Urology.* 2018;52(4):237-243.
31. Han DS, Zhou W, Seigne JD, Lynch KE, Schroeck FR. Geographic Variation in Cystoscopy Rates for Suspected Bladder Cancer between Female and Male Medicare Beneficiaries. *Urology.* 2018;122:83-88.

For peer review only - http://bmjopen.bmj.com/site/about/guidelines.xhtml

32. Garg T, Pinheiro LC, Atoria CL, et al. Gender disparities in hematuria evaluation and bladder cancer diagnosis: a population based analysis. *Journal of Urology.* 2014;192(4):1072-1077.
33. Murphy D, Meyer A, Vaghani V, et al. Application of Electronic Algorithms to Improve Diagnostic Evaluation for Bladder Cancer. *Appl Clin Inform.* 2017;8(1):279.
34. Ark JT, Alvarez JR, Koyama T, et al. Variation in the Diagnostic Evaluation among Persons with Hematuria: Influence of Gender, Race and Risk Factors for Bladder Cancer. *Journal of Urology.* 2017;198(5):1033-1038.
35. Bassett JC, Alvarez J, Koyama T, et al. Gender, race, and variation in the evaluation of microscopic hematuria among Medicare beneficiaries. *Journal of General Internal Medicine.* 2015;30(4):440-447.
36. Friedlander DF, Resnick MJ, You C, et al. Variation in the intensity of hematuria evaluation: a target for primary care quality improvement. *American Journal of Medicine.* 2014;127(7):633-640.e611.
37. Chappidi MR, Kates M, Tosoian JJ, et al. Evaluation of gender-based disparities in time from initial haematuria presentation to upper tract urothelial carcinoma diagnosis: analysis of a nationwide insurance claims database. *BJU International.* 2017;120(3):377-386.
38. Cohn JA, Vekhter B, Lyttle C, Steinberg GD, Large MC. Sex disparities in diagnosis of bladder cancer after initial presentation with hematuria: a nationwide claims-based investigation. *Cancer.* 2014;120(4):555-561.
39. Elias K, Svatek RS, Gupta S, Ho R, Lotan Y. High-risk patients with hematuria are not evaluated according to guideline recommendations. *Cancer.* 2010;116(12):2954-2959.
40. Lyratzopoulos G, Abel GA, McPhail S, Neal RD, Rubin GP. Gender inequalities in the promptness of diagnosis of bladder and renal cancer after symptomatic presentation: Evidence from secondary analysis of an English primary care audit survey. *BMJ Open.* 2013;3(e002861).
41. Ngo B, Papa N, Perera M, Bolton D, Sengupta S. Predictors of delay to cystoscopy and adequacy of investigations in patients with haematuria. *BJU International.* 2017;119 Suppl 5:19-25.
42. Richards KA, Ham S, Cohn JA, Steinberg GD. Urinary tract infection-like symptom is associated with worse bladder cancer outcomes in the Medicare population: Implications for sex disparities. *International Journal of Urology.* 2016;23(1):42-47.
43. Kelly JD, Fawcett DP, Goldberg LC. Assessment and management of non-visible haematuria in primary care. *BMJ: British Medical Journal (Online).* 2009;338(a3021).
44. Shapley M, Mansell G, Jordan JL, Jordan KP. Positive predictive values of $\geqslant$ 5% in primary care for cancer: systematic review. *Br J Gen Pract.* 2010;60(578):e366-e377.
45. Smittenaar C, Petersen K, Stewart K, Moitt N. Cancer incidence and mortality projections in the UK until 2035. *British Journal of Cancer.* 2016;115(9):1147-1155.
46. American Urological Association. Diagnosis, Evaluation and Follow-up of Asymptomatic Microhematuria (AMH) in Adults. 2012; http://www.auanet.org/guidelines/asymptomatic-microhematuria-(2012-reviewed-for-currency-2016). Accessed 1st September 2018.
47. Loo RK, Lieberman SF, Slezak JM, et al. Stratifying risk of urinary tract malignant tumors in patients with asymptomatic microscopic hematuria. *Mayo Clinic Proceedings.* 2013;88(2):129-138.

For peer review only - http://bmjopen.bmj.com/site/about/guidelines.xhtml

48. Tan WS, Ahmad A, Feber A, et al. Development and validation of a haematuria cancer risk score to identify patients at risk of harbouring cancer. *Journal of Internal Medicine.* 2018;doi: 10.1111/joim.12868.
49. Weingart SN, Stoffel EM, Chung DC, et al. Delayed workup of rectal bleeding in adult primary care: examining process-of-care failures. *The Joint Commission Journal on Quality and Patient Safety.* 2017;43(1):32-40.

For peer review only

For peer review only - http://bmjopen.bmj.com/site/about/guidelines.xhtml

For peer review only

For peer review only - http://bmjopen.bmj.com/site/about/guidelines.xhtml

Figure 1: PRISMA flow diagram

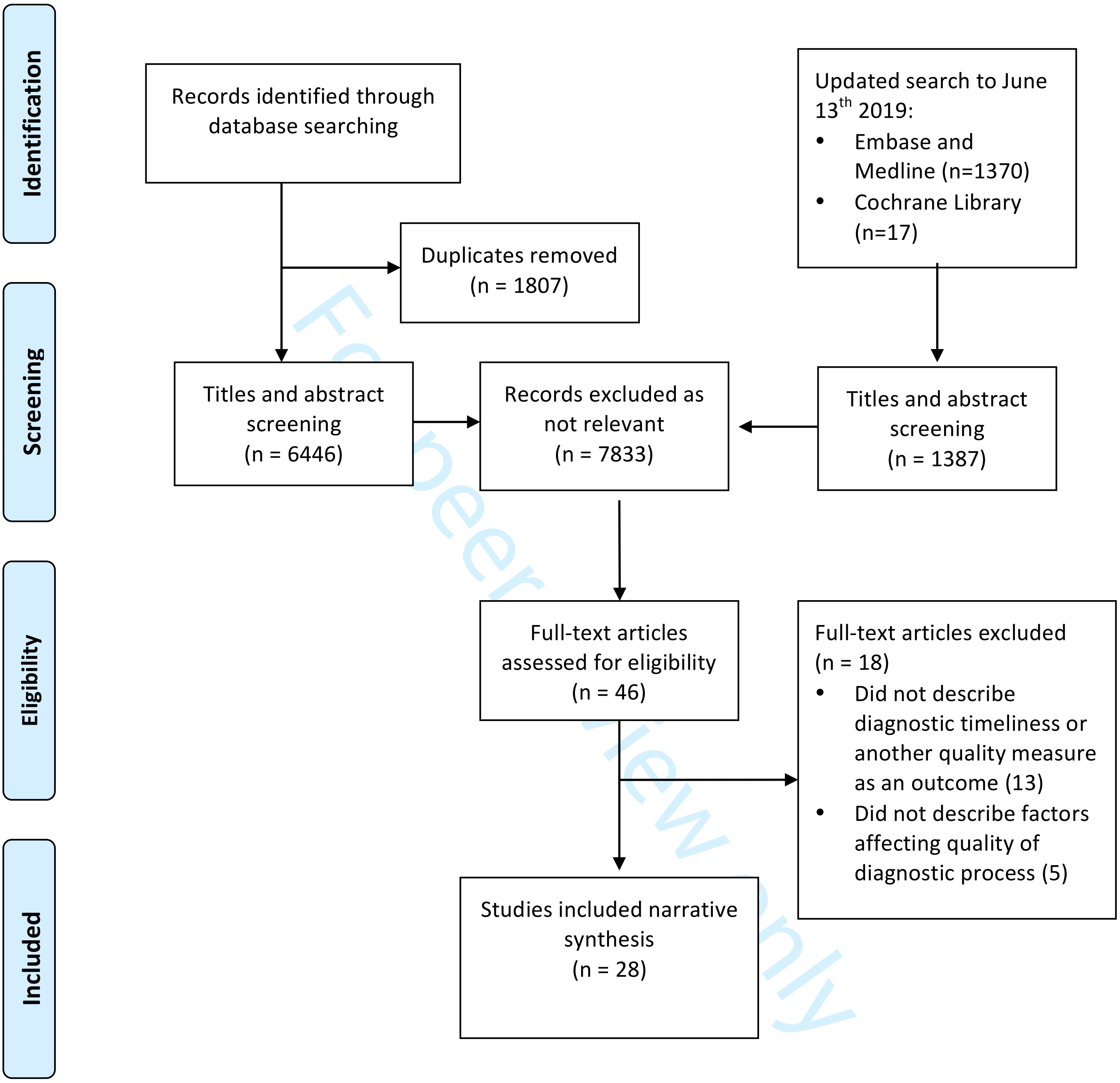

Appendix 1 – Example Search Strategy (1st January 2000 to 28th January 2018)

1. exp Urinary Tract Infections/ or exp lower urinary tract symptoms/ or exp hematuria/ or exp polyuria/ or exp nocturia/ or h?ematuri*.ti,ab. Or dysuri*.ti,ab. Or (nocturi* or nycturi*).ti,ab. Or polyuri*.ti,ab. Or exp Urinary Incontinence/ or (urinary adj3 incontinen*).ti,ab. Or (urin* adj3 (frequen* or urgen*)).ti,ab. Or (urin* adj3 infect*).ti,ab. – 379274
2. exp Kidney Neoplasms/ or ((Kidney or renal) adj3 (cancer or neoplas* or tumo?r or carcinoma*)).ti,ab. – 213088
3. exp Urinary Bladder Neoplasms/ or (bladder adj3 (cancer or neoplas* or tumo?r or carcinoma*)).ti,ab. – 145154
4. #2 OR #3 – 346524
5. #1 AND #4 – 20778
6. limit #5 to (humans and yr="2000-Current") – 12950
7. limit #7 to "all adult (19 plus years)" [Limit not valid in Embase; records were retained] – 12268
8. limit #8 to ("all adult (19 plus years)" or "adolescent (13 to 18 years)") [Limit not valid in Embase; records were retained] – 12268
9. limit #6 to (adult <18 to 64 years> or aged <65+ years>) [Limit not valid in Ovid MEDLINE(R),Ovid MEDLINE(R) Daily Update,Ovid MEDLINE(R) In-Process,Ovid MEDLINE(R) Publisher; records were retained] – 8253

Cochrane Library Search Strategy (inception to 13th June 2019)

#1	MeSH descriptor: [Kidney Neoplasms] explode all trees	1046
#2	MeSH descriptor: [Urinary Bladder Neoplasms] explode all trees	1356
#3	MeSH descriptor: [Diagnosis] explode all trees	315302
#4	MeSH descriptor: [Urinary Tract Infections] explode all trees	2370
#5	MeSH descriptor: [Lower Urinary Tract Symptoms] explode all trees	2777
#6	MeSH descriptor: [Hematuria] 2 tree(s) exploded	177
#7	#1 OR #2	2371
#8	#4 OR #5 OR #6	5223
#9	#7 AND #8	37
#10	#9 AND #3	17

For peer review only

Appendix 2: Reported percentages of diagnostic tests and urological referrals for patients with hematuria

Study	Type of hematuria*	No evaluation (%)	Frequency of different evaluations and referrals performed (%)							
			History/ Exam	Urine Culture	Urine Cytology	Imaging	Cystoscopy	Both Imaging and cystoscopy	Referral	Guideline concordant evaluation
**Performed within 30 days**										
**Matulewicz**	NVH					7.7	3.6	1.4		
**Performed within 60 days**										
**Richards 2018**	NVH	66								
**Richards 2018**	VH	67			-					
**Murphy**	VH+	47								
**Neider**	NVH			57-60	6-11	20-25			36	
**Performed within 90 days**										
**Richards 2018**	NVH	66								
**Richards 2018**	VH	47								
**Neider**	VH			40-56	5-13	38-41			69-77	
**Performed within 180 days**										
**Bassett**	NVH	65						14	23	
**Matulewicz**	NVH					14.3	8.9	5.1		
**Ark**	Either	54				44	20	18	35	
**Shinagare**	Either	36			43	76	35	64		36‡
**Friedlander**	Either					13.9	13.7	5.7		
**Performed within 365 days**										
**Matulewicz**	NVH					16.5	9.8	5.9		
**Performed within unspecified timescale (1-3 years)**										
**Elias**	NVH	42		15	10	23			21	
**Buteau**	Either	16		63	7.3					
**Bradley**	NVH		63	25						89§

For peer review only

**(secondary care)**										
**Buteau**	NVH			59	5	34	6	5		
**Buteau**	VH			84	20	42	26	25		
**Matulewicz**	NVH					6.2	3.0	2.5		

[*]NVH: non-visible hematuria; VH: visible hematuria
[+]Murphy included patients with 50 red blood cell per high power field (RBC/HPF), equivalent to very high risk and VH group
Definition of guideline concordant evaluation:
[‡] Shinagare: upper renal tract imaging, urine cytology and cystoscopy for high risk patients and 1 urinalysis with >3 RBC/HPF; or upper renal tract imaging followed by either urine cytology or cystoscopy for patients with no risk factors and 2 of 3 urinalysis with >3RBC/HPF.
[§] Bradley: cystoscopy and upper tract imaging using CT urography

For peer review only

PRISMA 2009 Checklist

Section/topic	#	Checklist item	Reported on page #
**TITLE**			
Title	1	Identify the report as a systematic review, meta-analysis, or both.	1
**ABSTRACT**			
Structured summary	2	Provide a structured summary including, as applicable: background; objectives; data sources; study eligibility criteria, participants, and interventions; study appraisal and synthesis methods; results; limitations; conclusions and implications of key findings; systematic review registration number.	2-3
**INTRODUCTION**			
Rationale	3	Describe the rationale for the review in the context of what is already known.	5
Objectives	4	Provide an explicit statement of questions being addressed with reference to participants, interventions, comparisons, outcomes, and study design (PICOS).	5-6
**METHODS**			
Protocol and registration	5	Indicate if a review protocol exists, if and where it can be accessed (e.g., Web address), and, if available, provide registration information including registration number.	NA
Eligibility criteria	6	Specify study characteristics (e.g., PICOS, length of follow-up) and report characteristics (e.g., years considered, language, publication status) used as criteria for eligibility, giving rationale.	7-8
Information sources	7	Describe all information sources (e.g., databases with dates of coverage, contact with study authors to identify additional studies) in the search and date last searched.	7
Search	8	Present full electronic search strategy for at least one database, including any limits used, such that it could be repeated.	Appendix 1
Study selection	9	State the process for selecting studies (i.e., screening, eligibility, included in systematic review, and, if applicable, included in the meta-analysis).	7-8
Data collection process	10	Describe method of data extraction from reports (e.g., piloted forms, independently, in duplicate) and any processes for obtaining and confirming data from investigators.	8
Data items	11	List and define all variables for which data were sought (e.g., PICOS, funding sources) and any assumptions and simplifications made.	8
Risk of bias in individual studies	12	Describe methods used for assessing risk of bias of individual studies (including specification of whether this was done at the study or outcome level), and how this information is to be used in any data synthesis.	8
Summary measures	13	State the principal summary measures (e.g., risk ratio, difference in means).	9(narrative)
Synthesis of results	14	Describe the methods of handling data and combining results of studies, if done, including measures of consistency (e.g., I^2) for each meta-analysis.	NA

For peer review only - http://bmjopen.bmj.com/site/about/guidelines.xhtml

PRISMA 2009 Checklist

Section/topic	#	Checklist item	Reported on page #
Risk of bias across studies	15	Specify any assessment of risk of bias that may affect the cumulative evidence (e.g., publication bias, selective reporting within studies).	8
Additional analyses	16	Describe methods of additional analyses (e.g., sensitivity or subgroup analyses, meta-regression), if done, indicating which were pre-specified.	NA
**RESULTS**			
Study selection	17	Give numbers of studies screened, assessed for eligibility, and included in the review, with reasons for exclusions at each stage, ideally with a flow diagram.	Figure 1
Study characteristics	18	For each study, present characteristics for which data were extracted (e.g., study size, PICOS, follow-up period) and provide the citations.	Table 1
Risk of bias within studies	19	Present data on risk of bias of each study and, if available, any outcome level assessment (see item 12).	Table 1
Results of individual studies	20	For all outcomes considered (benefits or harms), present, for each study: (a) simple summary data for each intervention group (b) effect estimates and confidence intervals, ideally with a forest plot.	Tables 2-4
Synthesis of results	21	Present results of each meta-analysis done, including confidence intervals and measures of consistency.	NA
Risk of bias across studies	22	Present results of any assessment of risk of bias across studies (see Item 15).	Table 1 and page 10
Additional analysis	23	Give results of additional analyses, if done (e.g., sensitivity or subgroup analyses, meta-regression [see Item 16]).	NA
**DISCUSSION**			
Summary of evidence	24	Summarize the main findings including the strength of evidence for each main outcome; consider their relevance to key groups (e.g., healthcare providers, users, and policy makers).	24-28
Limitations	25	Discuss limitations at study and outcome level (e.g., risk of bias), and at review-level (e.g., incomplete retrieval of identified research, reporting bias).	28
Conclusions	26	Provide a general interpretation of the results in the context of other evidence, and implications for future research.	29
**FUNDING**			
Funding	27	Describe sources of funding for the systematic review and other support (e.g., supply of data); role of funders for the systematic review.	30

From: Moher D, Liberati A, Tetzlaff J, Altman DG, The PRISMA Group (2009). Preferred Reporting Items for Systematic Reviews and Meta-Analyses: The PRISMA Statement. PLoS Med 6(6): e1000097. doi:10.1371/journal.pmed1000097

For peer review only - http://bmjopen.bmj.com/site/about/guidelines.xhtml

For more information, visit: www.prisma-statement.org.

PRISMA 2009 Checklist

For peer review only
